# Involvement of the reticulospinal system in the control of locomotion

**DOI:** 10.1113/JP290941

**Published:** 2026-09-03

**Authors:** Nicole Sarah Holliger, Volker Dietz, Céline Grünenfelder, Marina Geissmann, Linard Filli

**Affiliations:** ^1^ Spinal Cord Injury Center Balgrist University Hospital, University of Zurich Zurich Switzerland; ^2^ Department of Health Sciences and Technology ETH Zurich Zurich Switzerland; ^3^ Neuroscience Center Zurich University of Zurich Zurich Switzerland; ^4^ Swiss Center for Movement Analysis (SCMA), Balgrist Campus AG Zurich Switzerland; ^5^ Swiss Paraplegic Research Nottwil Switzerland

**Keywords:** brainstem, locomotor co‐ordination, neural limb coupling, reticulospinal control, startle reflex

## Abstract

**Abstract:**

Human stepping movements are known to be co‐ordinated by a neural coupling of upper and lower limbs. The aim of this study was to evaluate a potential involvement of the reticulospinal system in the interlimb co‐ordination by neural coupling during locomotion. During stepping electrical stimuli (ES) were applied to the right tibial nerve to assess the mechanisms underlying neural coupling of lower limbs, and loud acoustic stimuli (LAS) were released, known to activate the reticulospinal system. EMG activity of the tibialis anterior (TA) and gastrocnemius medialis (GM) of both sides was recorded during the right swing and double support phases of the step cycle and during dorsi‐ and plantarflexion movements of the feet. In all motor tasks the responses to both ES and LAS were modulated in the same way. The largest responses to both stimuli appeared during the right swing in the left GM and during double support in the left TA. During the right swing the left GM contributes to maintain body equilibrium over the left leg. In the double support phase the TA has to secure foot clearance for swing initiation. There was an absolute time shift in latency between responses to both ES and LAS across all tasks, compatible with a common neural structure for their generation in the brainstem. These findings suggest that reticulospinal drive is dynamically modulated and is involved in interlimb co‐ordination during locomotion.

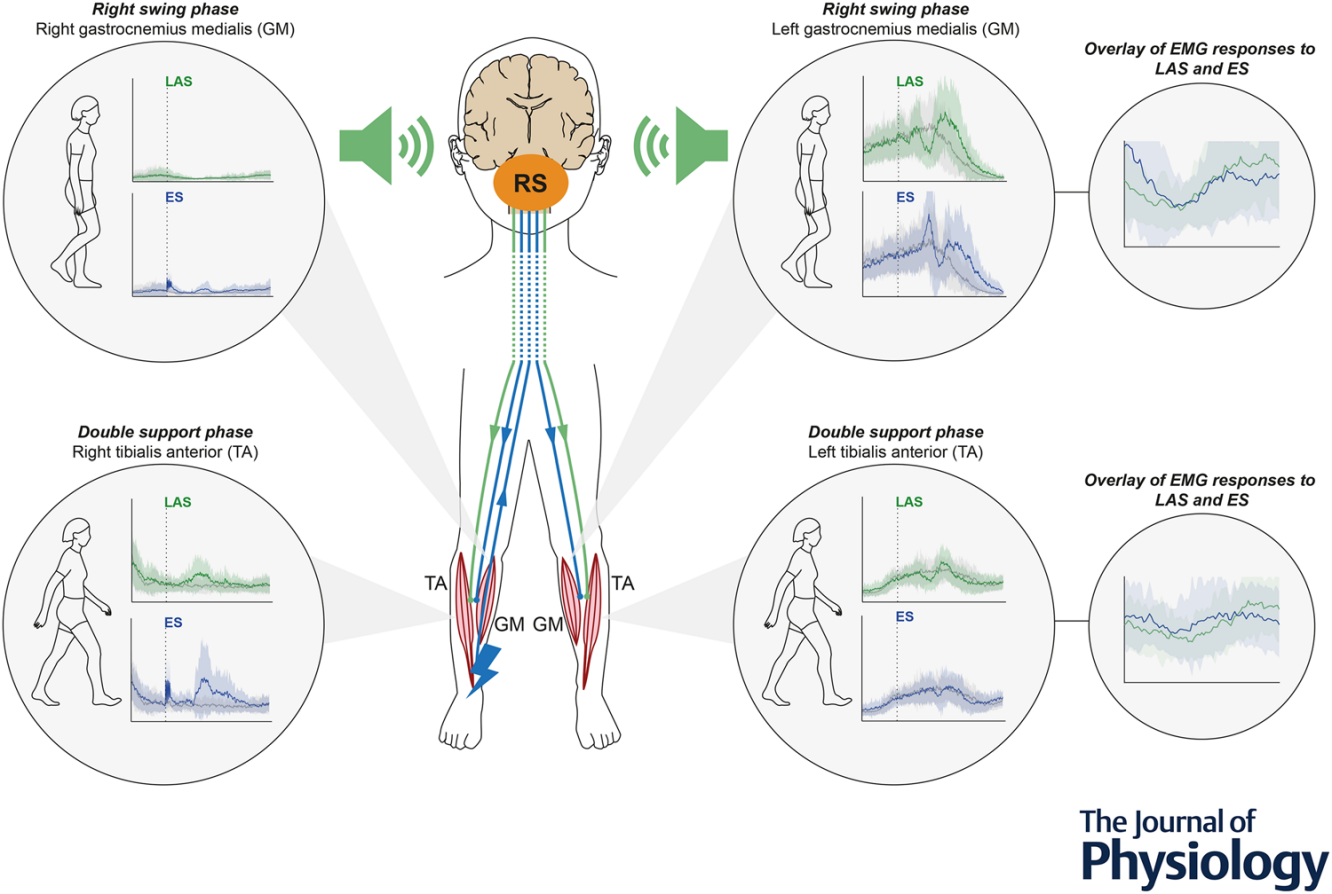

**Key points:**

- Unilateral electrical stimuli (ES), applied to the right tibial nerve, were used to explore the neural coupling mechanism and its contribution to co‐ordination of the legs during stepping.
- Loud acoustic stimuli (LAS) were applied to assess the potential involvement of the reticulospinal system in locomotor co‐ordination.
- LAS and ES evoked similar EMG responses in the left GM during the right swing and in the left tibialis anterior (TA) during double support.
- A 30 ms time shift between responses to LAS and ES across all tasks is compatible with a common origin of their generation in the brainstem.
- Indirect evidence suggests that neural leg coupling during stepping is mediated by brainstem structures, likely the reticulospinal system.

## Graphical abstract

**Abstract figure legend** During human locomotion the legs appear to be co‐ordinated by a neural coupling mechanism. This is reflected by the observation that electrical stimuli (ES) applied to the right tibial nerve during the right swing result in a neural coupling response in the left gastrocnemius (GM, blue EMG signal). Likewise neural coupling responses appear in the left tibialis anterior (TA, blue EMG signal) during double support. Similar responses (i.e. coupling‐like responses, green EMG signals) appear after the application of loud acoustic stimuli (LAS), that are known to activate reticulospinal neurons. The responses to LAS and ES show a constant time shift of 30 ms, aligning with a reticulospinal contribution to the responses. These findings support the hypothesis that the reticulospinal system is involved in the control of locomotion by neural coupling underlying leg co‐ordination.

## Introduction

Locomotor activity is known to be generated by the central pattern generator (CPG) within the thoracolumbar spinal cord in vertebrates (Grillner, 1975; Grillner & El Manira, 2020) and humans (Dietz, 1992). The programmed pattern of leg muscle activation is dynamically modulated by supraspinal control and multisensory proprioceptive afferents based on external requirements (Rossignol et al., 2006; Santuz et al. 2022). The basic co‐ordination of the locomotor pattern seems to be conserved across species. In humans this is reflected in the quadrupedal organization of bipedal gait, where arm swing is part of locomotion (Dietz, 2002). To date the way in which supraspinal motor centres control and modulate human locomotor co‐ordination remains open.

Supraspinal commands are crucial for initiating and modulating the stepping pattern based on environmental requirements. Stereotypic walking is thought to be primarily controlled by brainstem–spinal projections (Dubuc et al., 2023). Compelling evidence from animal experiments highlights the critical importance of the reticulospinal system in regulating locomotion (Grillner & El Manira, 2020; Hägglund et al., 2010). Reticulospinal neurons, in turn, receive locomotor commands from the mesencephalic locomotor region that control brainstem–spinal pathways essential for locomotion (Brocard et al., 2010; Noga et al., 2003). Under challenging balance conditions supraspinal drive from the vestibulospinal system plays a crucial role in regulating both postural and dynamic stability (Bent et al., 2005). More complex aspects of locomotion, such as anticipatory control (e.g. overcoming obstacles) and skilled, targeted locomotion, seem to critically rely on corticospinal commands during specific phases of the step cycle (Meyer et al., 2020; Schubert et al., 1999). Thus although accurate locomotor performance relies on commands from different supraspinal motor centres, reticulospinal drive seems to be particularly important for locomotor control. However evidence for the critical involvement of the reticulospinal system in locomotion is derived predominantly from preclinical studies.

An essential basis of automatic interlimb co‐ordination during locomotion represents neural coupling of the legs (Balter & Zehr, 2007; Dietz et al., 2001). This coupling mechanism is evidenced by the observation that unilateral electrical leg nerve stimulation elicits EMG responses in muscles of all four limbs (Dietz et al., 2001; Wannier et al., 2001). Neural coupling also appears to be involved in the co‐ordination of other complex movements, such as bilateral hand co‐ordination (Köchli et al., 2020). Neural structures potentially involved in the neural coupling are homologous cortical structures connected by the corpus callosum (Carson et al., 2004; Eliassen et al., 2000) or corticospinal projections from the hemispheres (i.e. ipsi‐ and contralateral corticospinal pathways). Alternatively the reticulospinal system is known to provide widespread motor drive to inter‐ and motoneurons of both sides of the spinal cord (Brocard et al., 2010; Davidson & Buford, 2004; Drew & Rossignol, 1990). In fact the reticulospinal system was suggested to be involved in the control of bilateral complex movements in non‐human primates (Baker, 2011; Riddle et al., 2009). Accordingly a recent study provided compelling evidence for a critical involvement of the reticulospinal system in interlimb co‐ordination during bilateral hand movements in humans (Dietz et al., 2024). The reticulospinal system might also contribute to interlimb co‐ordination during human locomotion.

An involvement of the reticulospinal system in human movement control can be studied by the application of loud acoustic stimuli (LAS), known to activate reticulospinal neurons (Dietz et al., 2024; Fisher et al., 2012). LAS can elicit brainstem‐related motor response, including auditory vestibular‐evoked responses (Ashford et al., 2016), and startle responses mediated by reticulospinal neurons (Brown et al., 1991). Additionally LAS also elicits other responses associated with the reticulospinal system, including the StartReact effect (Neumann et al., 2025; Tapia et al., 2022). LAS were shown to suppress neural activity in the motor cortex 30–60 ms after tone application (Furubayashi et al., 2000; Tapia et al., 2022). In the present study LAS were used to assess the involvement of the reticulospinal system in neural coupling underlying interlimb co‐ordination. Previous studies applying LAS during gait provided evidence for a phasic modulation of reticulospinal drive (Nieuwenhuijzen et al., 2000; Schepens & Delwaide, 1995).

In the present study unilateral electrical stimuli (ES) were applied to the right tibial nerve to assess neural interlimb coupling during stepping. In addition LAS were released to evaluate a possible involvement of the reticulospinal system in this neural coupling. The two different stimuli were applied during two critical phases of the step cycle and, for comparison, during ankle movements in a sitting position to probe the mechanisms of neural interlimb coupling.

It is hypothesized that leg muscle EMG responses to LAS, reflecting the activation of the reticulospinal system, and unilateral ES, reflecting neural limb coupling, are modulated in a similar way during the step cycle. A similar behaviour of the responses might indicate an involvement of the reticulospinal system in the interlimb co‐ordination during locomotion.

## Methods

### Participants

Nineteen healthy participants (13 females, age (means ± SD): 22.8 ± 1.7 years) were included in this study. Only participants without orthopaedic, neurological or hearing impairments were included. Written informed consent was obtained from all participants. The study was approved by the Ethics Committee of the Canton Zurich (Study‐ID: 2021‐00973) and registered online (clinicaltrials.gov; NCT04967274). The study was conducted according to the guidelines of the Declaration of Helsinki and Good Clinical Practice.

### Experimental set‐up

The study consisted of two experimental tasks, an ‘ankle’ task and a ‘walking’ task. The order of the tasks was pseudo‐randomized for each participant. Two different stimuli (i.e. LAS and ES) were applied during defined phases of the movements using the GRAIL virtual reality system (Motek Medical B.V., CL Houten, the Netherlands) that is able to integrate motion capture data in real time. LAS were applied to activate reticulospinal neurons and evoke reticulospinal drive (Tapia et al., 2022). Unilateral ES were applied to the distal part of the right tibial nerve to detect a neural ‘coupling response’, that is, an EMG response not only in the ipsilateral but also in the contralateral (left) leg muscles (Balter & Zehr, 2007; Dietz, 2002). To familiarize the participants with the stimuli a single LAS and ES were applied before the recordings. After familiarization a practice trial of the subsequent task was performed (i.e. ankle or walking task). An analogous practice trial was conducted for the second task.

#### Ankle task

**Figure 1:**
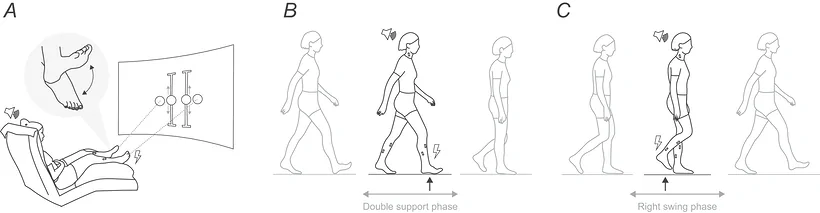
Schematic drawing of experimental set‐up *A*, participants performed visually guided, bilateral, dorsi‐ and plantarflexion foot movements, representing the ‘ankle task’. Participants moved their feet (outer circles) based on the displayed moving target paths (inner circles). LAS and ES (ES to the right tibial nerve) were randomly applied at defined time points either during dorsi‐ or plantarflexion feet movements. *B* and *C*, participants walked on a treadmill at 1 m/s. LAS and ES were randomly applied either (*B*) during the double support or (*C*) during the right swing phase. Vertical arrows indicate release of ES. LAS, loud acoustic stimuli; ES, electrical stimuli.

For the ankle task participants were comfortably sitting in a recliner and performed visually guided, bilateral and synchronous dorsi‐ and plantarflexion movements (Fig. 1*A*). The feet were positioned to allow full ankle range of motion (ROM). Movement trajectories were displayed to the participants using the GRAIL immersive virtual reality system. A marker‐based motion capture system (Vicon system, Oxford, UK) was coupled to the GRAIL system, allowing us to integrate real‐time motion data of the moving feet into the virtual environment. Three reflective markers, placed on the head of the fibula (knee), lateral malleolus (ankle) and head of the fifth metatarsal (toe) of each leg, were used to assess ankle ROM. Limits of the ankle movements were set to 90% of maximal ankle dorsi‐ and plantarflexion. Participants were instructed to follow the displayed targets with both feet synchronously for 140 s, resulting in 35 dorsi‐ and plantarflexion cycles per block. Additional resistance was applied to increase muscle activation during the dorsiflexion task (0.5 kg weight cuffs attached to the forefeet) and the plantarflexion task (elastic band connecting the forefeet with the torso, target force: 10 kg, measured using GS trainer (Gatherer Systems, Suffolk, UK)). LAS and ES were released at 80% of dorsi‐ or plantarflexion movement range. Two experimental blocks were performed for each task in a pseudo‐randomized order. Each task included 12 LAS and 12 ES. Each block contained 11 ‘dummy’ stimuli (no LAS or ES) that were used as baseline EMG activity. Between blocks breaks of 3–4 min were taken to prevent muscle fatigue.

#### Walking task

Participants walked on the treadmill at 1 m/s within a virtual forest environment presented by the GRAIL. LAS and ES were applied either during double support phase (Fig. 1*B*) or swing phase of the right leg (Fig. 1*C*). To ensure accurate stimulus triggering within the gait cycle, baseline stride, stance and double support times were calculated during the initial 15 s (after target speed was reached) of each assessment block when no stimuli were released. Heel strikes and toe offs were defined according to Zeni et al. (2008) and assessed by motion capture using a Plug in Gait marker set containing 36 markers.

Stimuli applied during the right swing phase were released after right toe off and targeting right mid‐swing phase. After detecting right heel strike, a trigger was released based on the following calculation: median baseline stance time (0.8 ± 0.1 s/67.5 ± 0.9% gait cycle) + 0.1 × median baseline stride time (1.2 ± 0.1 s). Stimuli applied during the double support phase were released at 50% of right double support (i.e. between right heel contact and left toe off). The trigger release after detecting right heel strike was based on the following calculation: median baseline stride time + 0.5 × baseline double support time (0.4 ± 0.0 s/34.7 ± 2.1% gait cycle).

Overall four experimental blocks were recorded. In two blocks the stimuli were applied during the swing phase, and in the other two blocks stimuli were applied during the double support phase. The order of the four blocks was pseudo‐randomized for each participant. Twelve LAS and 12 ES per block were applied in a pseudo‐randomized order. Baseline EMG activity was determined by recordings of 12 gait cycles where no stimuli were applied. Between blocks participants rested for 3–4 min to prevent muscle fatigue.

### Electromyographic recording

EMG activity was recorded using bipolar Ag‐AgCl surface EMG electrodes (H124SG, Kendall) positioned over the sternocleidomastoid (SCM), tibialis anterior (TA) and gastrocnemius medialis (GM) of both sides. Electrodes were placed according to SENIAM guidelines (Hermens et al., 1999) where applicable. EMG signals were recoded using a wireless EMG system (WaveX Picolite, Cometa Systems, Bareggio, Italy) with a sampling frequency of 1000 Hz. EMG data were bandpass‐filtered (10–499 Hz) and rectified using a customized Matlab script (Matlab R2022b, Mathworks Inc., MA, Natick, USA). No offset correction or normalization of the EMG recordings was performed.

### Acoustic stimulation

LAS (120 dB, 50 ms, 1000 Hz) were generated using a custom‐written Simulink script (Matlab 2021b), which was triggered by the D‐flow software of the GRAIL virtual reality system. LAS were presented through headphones (NTH‐100, Rode, Sydney, NSW, Australia). Sound intensity was calibrated before every measurement using a sound level meter (GRAS acoustics, Holte, Denmark).

### Electrical tibial nerve stimulation

Non‐noxious electrical stimuli were applied transcutaneously to the distal part of the right tibial nerve through self‐adhesive surface electrodes (Ambu A/S Neuroline 700, Denmark). Each stimulus consisted of five monophasic pulses (1 ms) separated by 2 ms. Stimuli were triggered using the D‐Flow software and released by an electrical current stimulator (Dantec KEYPOINT G4, Neurolite, Belp, Switzerland). Stimulation intensity was set to 150% of motor threshold defined as the first visible twitch of the first toe.

### Data analysis

Triggering time points of LAS and ES were recorded as analogue inputs in Vicon Nexus allowing data synchronization for the follow‐up analysis. Based on an earlier study EMG responses to LAS were defined as coupling‐like responses, consisting of a negative peak (N_CL_), followed by a positive peak (P_CL_) (Dietz et al., 2024). EMG responses to ES applied to the right tibialis nerve were defined as coupling responses, consisting of a negative (N_C_) and positive peak (P_C_). To assess the response modulations to LAS and ES compared to baseline activity, analysis of EMG response size was performed for leg muscles of both sides. However the analysis of coupling‐like and coupling responses focused on the EMG responses on the left side (contralateral to the stimulation side). The response size of the negative part of coupling‐like responses to LAS was calculated by the root mean square from 60 to 100 ms (RMS_60–100 ms_), indicating the first time window. For the positive part of the coupling‐like response its size was calculated from 100 to 140 ms (RMS_100–140 ms_), defined as the second time window. The coupling responses induced by ES appeared ∼30 ms later than the coupling‐like responses induced by LAS. Therefore the time windows for the analysis of response size were shifted by 30 ms relative to LAS‐evoked responses: the negative part was calculated from 90 to 130 ms (RMS_90–130 ms_), whereas the positive part was assessed from 130 to 170 ms (RMS_130–170 ms_). For comparison of the LAS‐ and ES‐evoked response sizes to baseline EMG activity the RMS time windows of baseline activity were matched to the above‐mentioned time windows used for the analysis of LAS and ES responses.

Negative‐to‐positive peak latencies of the rectified signal to LAS (N_CL_ and P_CL_) and ES (N_C_ and P_C_) were identified using a custom‐written Matlab script that detected local minima (from 60–150 ms) and maxima (from 100–190 ms) of the responses compared to baseline EMG activity. All identified local maxima and minima were visually inspected and manually corrected if necessary.

Startle responses to LAS and ES were present in the SCM. These were detected using a custom‐written Matlab script. A startle response was identified when the EMG signal exceeded the baseline value by >1 SD for at least 2 ms. Baseline EMG activity was calculated as the mean over 100 ms before stimulus onset. These automatically detected startle responses were visually inspected and manually corrected if necessary. Startle response latency was defined as the time interval between stimulus release and response onset.

### Statistical analysis

Statistical analysis was performed using R studio (R version 4.2.1/R Studio 2023.06.0 for windows) and Matlab. Statistics for the ankle task was based on 9 participants (10 participants were excluded due to a deficient stimulus‐triggering mechanism for both LAS and ES), whereas statistics for the walking task were based on all 19 participants. The level of significance was set at 0.05 for all statistical tests. All tests were adjusted for multiple comparisons using *post hoc* Holm‐Bonferroni correction. Normal distribution of data was assessed using the Shapiro‐Wilk test. Normal distributed data were assessed using parametric tests (paired *t* test, repeated‐measures one‐way ANOVA). Non‐normally distributed data were analysed using non‐parametric statistical tests (Wilcoxon matched pairs signed‐rank test, Friedman's test). Habituation of acoustic startle responses in the SCM over the four experimental blocks of each task (i.e. ankle and walking task) was assessed using Friedman's test. Difference in response size between LAS‐ and ES‐induced responses and baseline EMG activity of the two different time windows (i.e. RMS_60–100ms_ LAS *vs*. RMS_60–100ms_ baseline *vs*. RMS_90–130ms_ ES *vs*. RMS_90–130ms_ baseline; RMS_100–140ms_ LAS *vs*. RMS_100–140ms_ baseline *vs*. RMS_130–170ms_ ES *vs*. RMS_130–170ms_ baseline) was calculated using repeated‐measures one‐way ANOVA and Friedman's test with the following *post hoc* pairwise comparisons. Difference in response latencies between LAS and ES was assessed using paired *t* tests and Wilcoxon matched pairs signed‐rank tests. Comparison of LAS‐ES time shift of negative (N_C_–N_CL_) and positive response latencies (P_C_–P_CL_) was analysed using one‐way ANOVA and the Kruskal‐Wallis rank‐sum test.

Similarity of the EMG responses to LAS and ES was assessed using time series analysis. One‐dimensional statistical parametric mapping (SPM) was applied (Pataky et al., 2013) using the open‐source spm1d package (version 0.4, https://spm1d.org/) (Pataky, 2012). The normality of the data was confirmed using the spm1d built‐in function (spm1d.stats.normality.ttest_paired). As our data were not normally distributed, statistical non‐parametric mapping (SnPM) was performed (spm1d.stats.nonparam.ttest_paired). The null hypothesis was rejected if the SnPM {t} exceeded the critical threshold *t* ^∗^ at α = 0.05. Time series data included both negative and positive coupling‐like responses (N_CL_ and P_CL_). Therefore EMG responses to LAS included data from 60 to 140 ms after stimulus onset, and EMG responses to ES included data from 90 to 170 ms after stimulus onset. Additionally SPM analysis was used to detect a potential presence and habituation of startle responses to LAS and ES. For this purpose the time series data (60–140 ms (LAS) and 90–170 ms (ES)) were compared between the first six *vs*. the last six stimuli across all tasks.

## Results

### Startle responses in SCM and leg muscles

**Figure 2:**
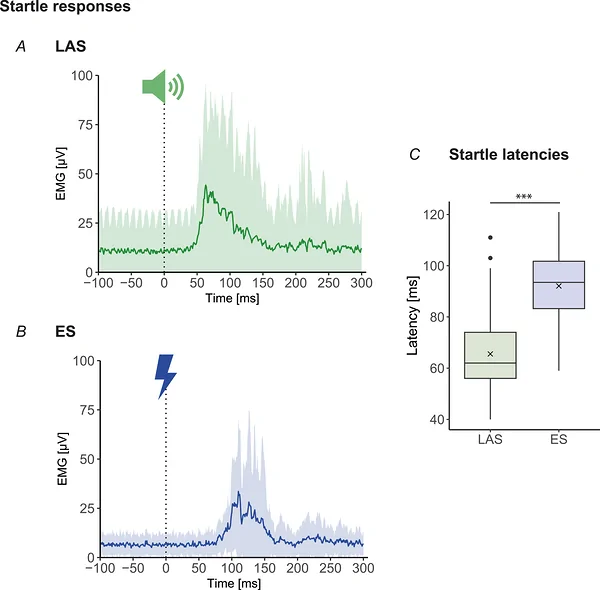
Startle responses to loud acoustic stimuli (LAS) and electrical stimuli (ES) *A*, LAS‐evoked startle responses in the SCM during the ankle and walking tasks. *B*, ES‐evoked startle responses in the SCM during the ankle and walking tasks. Vertical dotted lines represent the onset of the randomly released stimuli (LAS or ES). Graphs represent the mean of pooled startle responses (coloured lines) ± SD (shaded area). *C*, pooled onset latencies of startles elicited by LAS or ES during the ankle and walking tasks. Boxplots display median values (vertical black line) ± interquartile range (filled boxes) and mean values (black cross). Statistical differences are indicated by asterisks (^***^ *P* < 0.001). SCM, sternocleidomastoid.

LAS resulted in startle responses in the SCM (Fig. 2*A*) with an overall incidence of 9.1% across all tasks. There was a significant habituation of startle responses over the four experimental blocks in both tasks (ankle task: Friedman's test, Friedman's χ^2^ (3) = 13.033, *P* = 0.005, ω = 0.483; walking task: Friedman's test, Friedman's χ^2^ (3) = 26.439, *P* < 0.001, ω = 0.464). Startle responses in the SCM were also elicited using ES (Fig. 2*B*), however at a lower incidence (4.4%). No habituation of ES‐induced startle responses was observed (ankle task: Friedman's test, Friedman's χ^2^ (3) = 3.714, *P* = 0.294, ω = 0.065; walking task: Friedman's test, Friedman's χ^2^ (3) = 2, *P* = 0.572, ω = 0.074). Only five participants showed both LAS‐ and ES‐induced startle responses in the SCM. Therefore the comparison between LAS‐ and ES‐induced startle latencies was performed on pooled data. Latency of startle responses to LAS was (median ± interquartile range (IQR)) 62.0 ± 18.0 ms. Startle latency after ES was 93.5 ± 18.5 ms; that is it was significantly longer compared to LAS‐evoked startle responses (Mann–Whitney *U* test, *z* = −8.211, *P* <0.001, *r* = 0.615; Fig. 2*C*). The difference in latency between LAS‐ and ES‐ induced startle responses was (median ± IQR) 28.5 ± 11.5 ms. The latency difference was calculated based on the grand average of the five participants who showed startles to both LAS and ES.

In contrast to the SCM no startle responses were observed in muscles of the left leg (TA and GM). To examine the presence of potential startle responses in TA and GM habituation of EMG responses was investigated by comparing the first six *vs*. the last six EMG traces to LAS and ES. EMG responses were compared from 60 to 140 ms for LAS and from 90 to 170 ms for ES using SnPM. Overall there was no significant habituation of LAS‐ and ES‐induced responses in leg muscles across the different task in the lower leg muscles. Except for a single significant data point (163 ms) regarding ES‐induced responses in the left TA during double support phase there were no other data points exceeding t*< −3.715 across all comparisons.

### Leg muscle EMG responses during ankle joint movements

**Figure 3:**
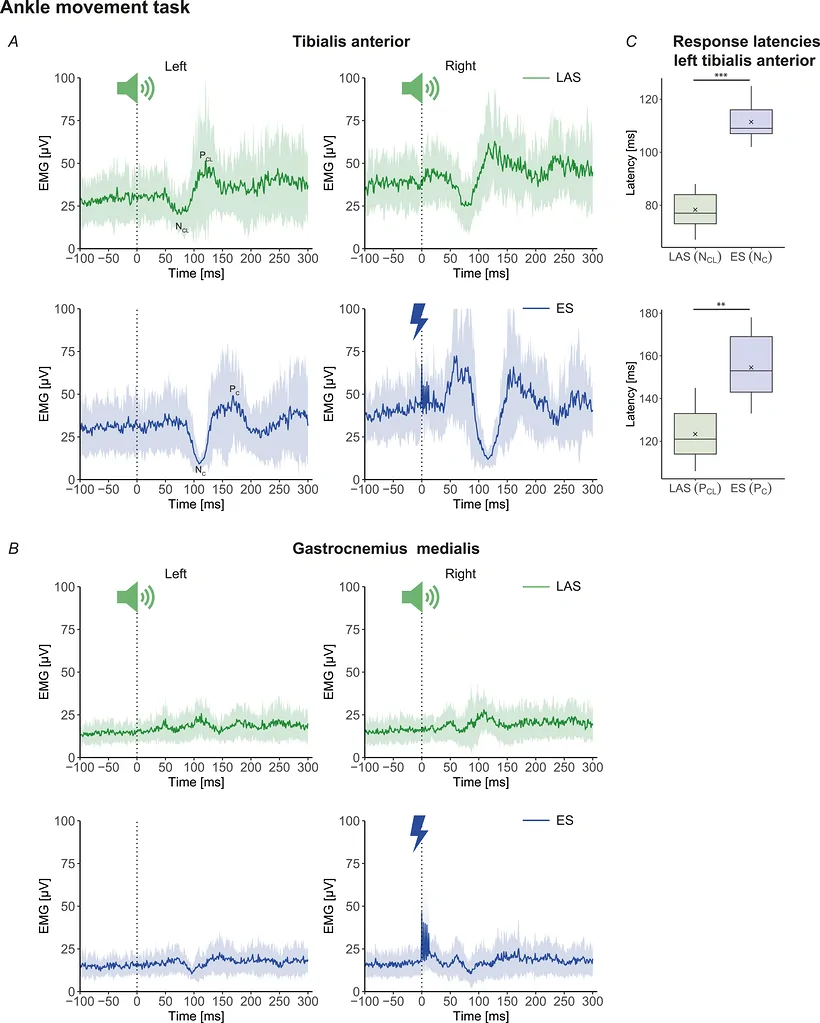
EMG responses to loud acoustic stimuli (LAS) and electrical stimuli (ES) during the ankle task *A*, EMG responses to LAS and ES in the left and right tibialis anterior (TA) during bilateral ankle dorsiflexion (Fig. 1). LAS resulted in coupling‐like responses (green line) in the left and right TA (N_CL_, negative peak; P_CL_, positive peak). Unilateral ES evoked coupling responses (blue line) (N_C_, negative peak; P_C_, positive peak) in the TA. There was an additional early peak in the right TA, ipsilateral to ES. *B*, EMG responses to LAS and ES in the left and right gastrocnemius medialis (GM) during plantarflexion. No distinct coupling‐like or coupling responses were elicited. Dotted lines indicate stimulation onset. Graphs display grand averages (coloured lines) ± SD (shaded area). *C*, latencies of LAS‐ and ES‐induced negative and positive response peaks in the left TA. Boxplots represent the median (black line) ± interquartile range (filled boxes) and mean values (black cross) of nine participants. Statistical differences are indicated by asterisks (^**^ *P* < 0.01, ^***^ *P* < 0.001).

During the dorsiflexion task coupling‐like and coupling responses were evoked. Within the first time window, during feet dorsiflexion, EMG activity to LAS and ES in the left TA differed significantly to baseline activity (*P* < 0.001; Table 1). The pairwise comparisons indicated a negative response modulation after LAS and ES compared to baseline (LAS: *P* = 0.035; ES: *P* < 0.001; Table 2). In contrast no significant positive response components were observed in the left TA in LAS‐ and ES‐induced responses relative to baseline activity (Fig. 3*A*; Tables 1 and 2). Although only significant negative response components were found following LAS and ES, negative‐to‐positive peak latencies after LAS (coupling‐like response) and ES (coupling response) were identified in all participants (Table 5). There was a significant time shift between LAS‐ and ES‐induced response latencies (N_CL_ *vs*. N_C_: *T* (8) = −14.032, *P* < 0.001, *r* = 0.541; P_CL_ *vs*. P_C_: T (8) = −3.547, *P* = 0.008, *r* = 0.666) (Table 5; Fig. 3*C*). For the right side a similar EMG response pattern to LAS and ES was observed. In the first time window EMG responses to LAS and ES differed from baseline (*P* < 0.001; Table 1). A negative response to LAS (*P* = 0.019; Table 2) and ES (*P* < 0.001; Table 2) appeared and differed from baseline (Fig. 3*A*). EMG responses that appeared on the right side were not further analysed, as only coupling‐like and coupling responses on the left side were of primary interest.

**Table 1:** Statistical analysis of response size across all tasks

Ankle task – tibialis anterior			
	*F*‐value(dfn, dfd)/χ^2^ value(df)	Cohen's *f*/Kendall's ω	*P*‐value
***First time window right***	**χ^2^ (3) = 22.467**	**ω = 0.832**	**< 0.001** ^***^
*Second time window right*	*F* (3,24) = 0.641	*F* = 0.283	0.596
***First time window left***	**χ^2^ (3) = 22.733**	**ω = 0.842**	**< 0.001** ^***^
*Second time window left*	χ^2^ (3) = 0.867	ω = 0.321	0.867

Ankle task – gastrocnemius medialis			
	*F‐*value(dfn, dfd)/χ^2^ value(df)	Cohen's *f*/Kendall's ω	*P*‐value
*First time window right*	*F* (1.93,15.45) = 0.641	*F* = 0.491	0.18
*Second time window right*	*F* (1.37,10.93) = 0.653	*F* = 0.285	0.483
*First time window left*	*F* (3,24) = 2.158	*F* = 0.519	0.119
***Second time window left***	***F* (3,24) = 5.441**	***F* = 0.825**	0.005^**^

Walking task – right swing phase – tibialis anterior			
	*F*‐value(dfn, dfd)/χ^2^‐value(df)	Cohen's *f*/Kendall's ω	*P*‐value
***First time window right***	**χ^2^ (3) = 24.284**	**ω = 0.426**	**< 0.001** ^***^
*Second time window right*	*F* (1.65,29.69) = 3.333	*F* = 0.430	0.058
*First time window left*	χ^2^ (3) = 1.421	ω = 0.025	0.701
***Second time window left***	**χ^2^ (3) = 24.032**	**ω = 0.422**	**< 0.001** ^***^

Walking task – right swing phase – gastrocnemius medialis			
	*F*‐value(dfn, dfd)/χ^2^ value(df)	Cohen's *f*/Kendall's ω	*P*‐value
***First time window right***	χ**^2^ (3) = 27.821**	**ω = 0.488**	**< 0.001** ^***^
*Second time window right*	χ^2^ (3) = 5.463	ω = 0.096	0.141
***First time window left***	**χ^2^ (3) = 15.821**	**ω = 0.278**	0.001^**^
***Second time window left***	**χ^2^ (3) = 38.937**	**ω = 0.683**	**< 0.001** ^***^

Walking task – double support phase – tibialis anterior			
	*F*‐value(dfn, dfd)/χ^2^ value(df)	Cohen's *f*/Kendall's ω	*P*‐value
***First time window right***	**χ^2^ (3) = 22.263**	**ω = 0.391**	**< 0.001** ^***^
***Second time window right***	***F* (1.65,29.783) = 10.677**	***F* = 0.770**	**< 0.001** ^***^
***First time window left***	**χ^2^ (3) = 17.97**	**ω = 0.315**	**< 0.001** ^***^
*Second time window left*	*F* (3,54) = 2.158	*F* = 0.346	0.104

Walking task – double support phase – gastrocnemius medialis			
	*F*‐value(dfn, dfd)/χ^2^ value(df)	Cohen's *f*/Kendall's ω	*P*‐value
***First time window right***	**χ^2^ (3) = 12.789**	**ω = 0.224**	**0.005** ^**^
***Second time window right***	**χ^2^ (3) = 11.27**	**ω = 0.198**	**0.01** ^*^
*First time window left*	χ^2^ (3) = 7.863	ω = 0.138	0.05
*Second time window left*	χ^2^ (3) = 0.411	ω = 0.007	0.938

*Note*: This table represents statistical comparisons of response components between loud acoustic stimuli (LAS)‐ and electrical stimuli (ES)‐induced responses and baseline activity. Analysis of the first time window includes the comparisons of the following response components: RMS_60–100ms_ of LAS‐induced responses, RMS_60–100ms_ of baseline activity, RMS_90–130ms_ of ES‐induced responses and RMS_90–130ms_ of baseline activity. Analysis of the second time window includes the comparisons of the following response components: RMS_100–140ms_ of LAS‐induced responses, RMS_100–140ms_ of baseline activity, RMS_130–170ms_ of ES‐induced responses and RMS_130–170ms_ of baseline activity. For the ANOVA *F*‐value and Cohen's *d* are reported. For the Friedman test χ^2^ values and Kendall's ω are reported.

Statistical differences are indicated in bold letters and by asterisks (^*^ *P* < 0.05, **P < 0.01, ***P < 0.001).

**Table 2:** EMG responses in tibialis anterior (TA) and gastrocnemius medialis (GM) of both sides during the ankle tasks

Tibialis anterior				
	Baseline LAS	LAS	Effect size	*P*‐value (pairwise comparison)
***RMS_60‐100ms_ right [µV]***	**51.0 ± 18.0**	**32.2 ± 14.1**	**0.909**	0.019^*^
*RMS_100‐140ms_ right [µV]*	51.2 ± 21. 2	54.5 ± 21.4	0.373	0.296
***RMS_60‐100ms_ left [µV]***	**40.9 ± 22.5**	**25.3 ± 14.2**	**0.84**	0.035^*^
*RMS_100‐140ms_ left [µV]*	41.9 ± 24.7	43.6 ± 28.4	0.151	1

	Baseline ES	ES	Effect size	*P*‐value (pairwise comparison)
***RMS_90‐130ms_ right [µV]***	**51.5 ± 19.8**	**25.9 ± 11.3**	**1.373**	< 0.001^***^
*RMS_130‐170ms_ right [µV]*	52.2 ± 20.9	50.9 ± 25.1	0.131	0.704
***RMS_90‐130ms_ left [µV]***	**42.3 ± 24.0**	**20.7 ± 10.3**	**1.466**	< 0.001^***^
*RMS_130‐170ms_ left [µV]*	41.5 ± 24.9	42.0 ± 25.4	0.151	1

Gastrocnemius medialis				
	Baseline LAS	LAS	Effect size	*P*‐value (pairwise comparison)
*RMS_60‐100ms_ right [µV]*	19.9 ± 9.0	19.0 ± 11.6	0.155	1
*RMS_100‐140ms_ right [µV]*	20.3 ± 9.4	23.0 ± 8.9	0.621	0.596
*RMS_60‐100ms_ left [µV]*	18.1 ± 7.1	17.8 ± 7.9	0.063	1
***RMS_100‐140ms_ left [µV]***	**16.1 ± 6.6**	**20.5 ± 8.2**	**1.205**	0.041^*^

	Baseline ES	ES	Effect size	*P*‐value (pairwise comparison)
*RMS_90‐130ms_ right [µV]*	20.2 ± 8.9	16.9 ± 7.8	0.843	0.212
*RMS_130‐170ms_ right [µV]*	20.7 ± 9.8	20.9 ± 11.5	0.017	1
*RMS_90‐130ms_ left [µV]*	18.0 ± 6.5	15.4 ± 6.2	0.96	0.123
*RMS_130‐170ms_ left [µV]*	17.9 ± 7.0	19.9 ± 8.9	0.85	0.137

*Note*: Loud acoustic stimuli (LAS)‐ and electrical stimuli (ES)‐induced responses were compared to baseline activity. Response components of the first time window is indicated by RMS_60–100ms_ for LAS‐induced responses and corresponding baseline activity, and by RMS_90–130ms_ for ES‐induced responses and corresponding baseline activity. Response components of the second time window are indicated by RMS_100–140ms_ for LAS‐induced responses and corresponding baseline activity, and by RMS_130–170ms_ for ES‐induced responses and corresponding baseline activity. TA was analysed during dorsiflexion and GM during plantarflexion. Data represent mean values ± SD from nine participants.

Statistical significances are indicated in bold letters and by asterisks (^*^ *P* < 0.05, **P < 0.01, ***P < 0.001).

During the plantarflexion task evoked responses in the left GM were generally modest and only appeared in the second time window (*P* = 0.005; Table 1). Pairwise comparison revealed that there was a positive response to LAS (*P* = 0.041; Table 2; Fig. 3*B*), whereas no further responses to ES and LAS were detected (Tables 1 and 2).

### Evoked EMG responses in leg muscles during walking

#### Right swing phase

**Figure 4:**
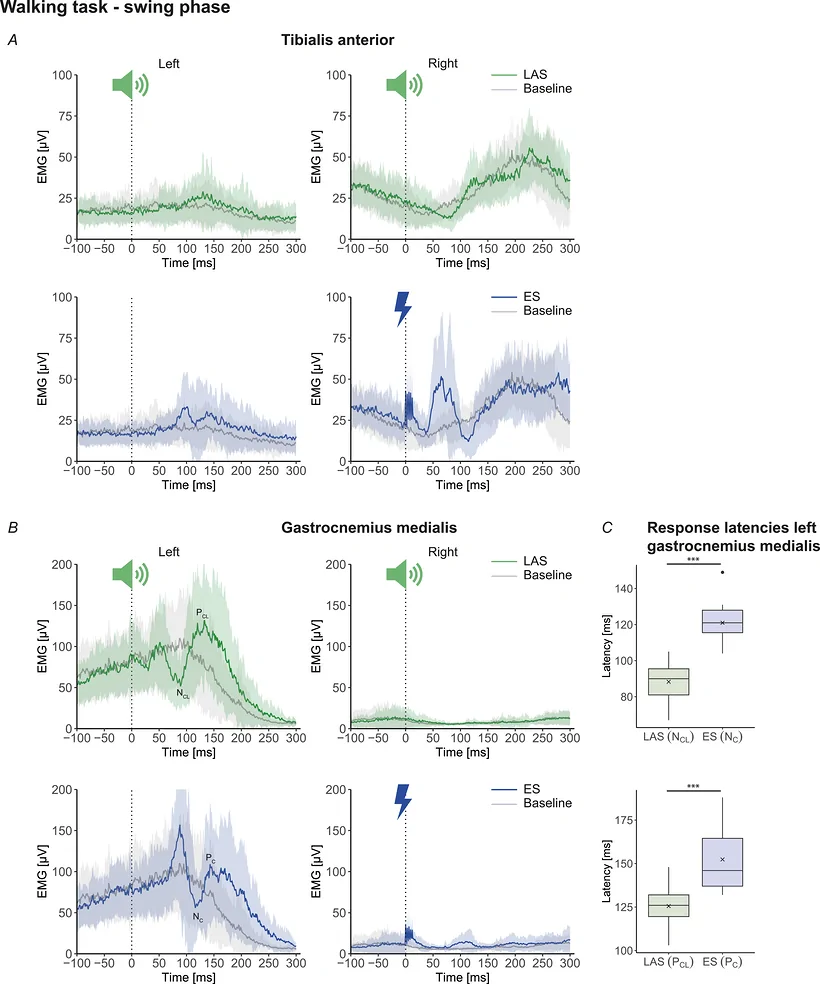
EMG responses to loud acoustic stimuli (LAS) and electrical stimuli (ES) during the right swing in the walking task *A*, EMG activity of the left and right tibialis anterior (TA) during the right swing phase. Grey traces indicate baseline EMG activity of trials in which no stimuli were applied. Green and blue traces indicate EMG activity of trials where LAS or ES were applied. *B*, EMG responses in the left and right gastrocnemius medialis (GM) during the right swing. Grey traces indicate baseline EMG activity of trials in which no stimuli were applied. Green (LAS) and blue (ES) traces indicate EMG activity of trials where LAS and ES were randomly released. Coupling‐like (LAS; negative peak (N_CL_) and positive peak (P_CL_)) and coupling responses (ES; N_C_, P_C_) appeared in the left GM to LAS and ES. Black vertical dotted lines represent onset of stimulation. Graphs indicate grand averages (coloured lines) ± SD (shaded area) of all 19 participants. *C*, latencies of LAS‐ and ES‐induced responses in the left GM. Boxplots display the median (black line) ± interquartile range (filled boxes) and mean values (black cross) of the negative and positive peak latencies. Statistical differences are indicated by asterisks (^***^ *P* < 0.001).

In the TA during the right swing phase no clear neural coupling responses were present. There was no negative response in the left TA in the first time window (Table 1). However LAS‐ and ES‐induced EMG responses differed from baseline activity in the second time window (*P* < 0.001; Table 1). There was a positive response modulation to LAS (*P* = 0.016; Table 3) and ES (*P* < 0.001; Table 3) compared to baseline (Fig. 4*A*). As negative‐to‐positive response peak latencies only emerged in 26.3% of all participants, no further analysis of coupling‐like and coupling responses was performed. In the right TA only the response component in the first time window revealed a significant difference between LAS‐ and ES‐induced responses and baseline activity (*P* < 0.001; Table 1). *Post hoc* analysis showed that LAS induced a negative response that differed from baseline activity (*P* = 0.02; Table 3; Fig. 4*A*).

**Table 3:** EMG responses in the left and right tibialis anterior (TA) and gastrocnemius medialis (GM) during the right swing in the walking task

Tibialis anterior				
	Baseline LAS	LAS	Effect size	*P*‐value (pairwise comparison)
***RMS_60‐100ms_ right [µV]***	**22.3 ± 8.9**	**16.1 ± 6.8**	**0.659**	0.02^*^
*RMS_100‐140ms_ right [µV]*	27.9 ± 8.3	32.7 ± 11.8	0.563	0.123
*RMS_60‐100ms_ left [µV]*	20.8 ± 10.3	20.4 ± 9.5	0.014	1
***RMS_100‐140ms_ left [µV]***	**20.6 ± 11.8**	**25.6 ± 14.8**	**0.658**	0.016^*^

	Baseline ES	ES	Effect size	*P*‐value (pairwise comparison)
*RMS_90‐130ms_ right [µV]*	25.8 ± 8.6	21.8 ± 11.1	0.538	0.057
*RMS_130‐170ms_ right [µV]*	36.7 ± 12.1	34.4 ± 17.0	0.158	1
*RMS_90‐130ms_ left [µV]*	20.4 ± 11.0	29.2 ± 19.1	0.243	1
***RMS_130‐170ms_ left [µV]***	**19.7 ± 12.3**	**27.2 ± 14.7**	**0.914**	< 0.001^*^

Gastrocnemius medialis				
	Baseline LAS	LAS	Effect size	*P*‐value (pairwise comparison)
*RMS_60‐100ms_ right [µV]*	6.0 ± 1.6	6.0 ± 2.0	0.186	0.417
*RMS_100‐140ms_ right [µV]*	6.6 ± 1.8	7.0 ± 3.1	0.072	1
***RMS_60‐100ms_ left [µV]***	**102.5 ± 51.1**	**71.9 ± 34.0**	**0.914**	< 0.001^***^
***RMS_100‐140ms_ left [µV]***	**88. 3 ± 54.5**	**115.8 ± 59.5**	**0.784**	0.003^**^

	Baseline ES	ES	Effect size	*P*‐value (pairwise comparison)
*RMS_90‐130ms_ right [µV]*	6.4 ± 1.7	14.1 ± 9.8	0.538	0.057
*RMS_130‐170ms_ right [µV]*	7.7 ± 3.5	9. 2 ± 4.4	0.186	1
*RMS_90‐130ms_ left [µV]*	94.9 ± 55.7	95.2 ± 69.1	0.072	0.755
***RMS_130‐170ms_ left [µV]***	**65.7 ± 49.0**	**101.1 ± 51.9**	**0.981**	< 0.001^***^

*Note*: Loud acoustic stimuli (LAS)‐ and electrical stimuli (ES)‐induced responses were compared to baseline activity. Response component in the first time window is indicated by RMS_60–100ms_ for LAS‐induced responses and corresponding baseline activity, and by RMS_90–130ms_ for ES‐induced responses and corresponding baseline activity. Response component in the second time window is indicated by RMS_100–140ms_ for LAS‐induced responses and corresponding baseline activity, and by RMS_130–170ms_ for ES‐induced responses and corresponding baseline activity. Data represent mean values ± SD from 19 participants.

Statistical differences are indicated in bold letters and by asterisks (^*^ *P* < 0.05, **P < 0.01, ***P < 0.001).

In the left GM characteristic coupling‐like and coupling responses were identified. LAS and ES elicited EMG responses that differed significantly from baseline activity in both time windows (first time window: *P* = 0.001; second time window: *P* < 0.001; Table 1). *Post hoc* analysis revealed that LAS induced a negative (*P* < 0.001; Table 3) and a positive response modulation (*P* = 0.003; Table 3) compared to baseline. ES evoked responses in which the positive component differed significantly from baseline (*P* < 0.001; Table 3; Fig. 4*B*). LAS and ES evoked negative‐to‐positive peak latencies in all participants. Coupling responses to ES were shifted in latency compared to the coupling‐like response (N_CL_ *vs*. N_C_: T (18) = −12.737, *P* < 0.001, *r* = 0.479; P_CL_ *vs*. P_C_: Z = −3.763, *P* < 0.001, *r* = 0.303; Table 5; Fig. 4*C*). In the right GM the responses to LAS and ES differed significantly from baseline activity in the first time window (*P* < 0.001; Table 1) but not in the second time window. However pairwise comparisons revealed no significant differences (Table 3; Fig. 4*B*).

#### Double support phase

**Figure 5:**
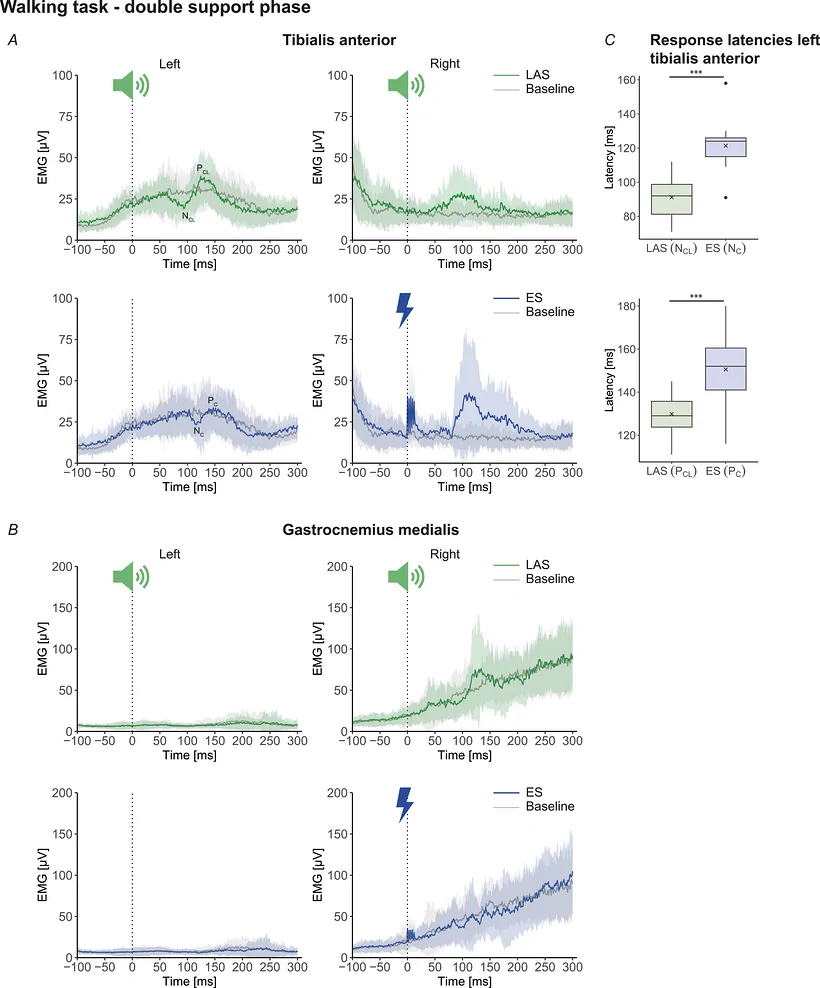
EMG responses to loud acoustic stimuli (LAS) and electrical stimuli (ES) during the double support in the walking task *A*, EMG activity of the left and right tibialis anterior (TA) during the double support. Overlay of baseline EMG activity (grey lines) and EMG responses to LAS (green line) and ES (blue line). Coupling‐like (N_CL_, P_CL_) and coupling responses (NC, PC) occurred in the left TA after both LAS and ES. *B*, EMG activity of the left and right gastrocnemius medialis (GM) during the double support. Grey traces indicate baseline EMG activity of trials in which no stimuli were applied. Green (LAS) and blue (ES) traces indicate EMG responses of trials where LAS and ES were randomly released. Black vertical dotted lines indicate onset of stimulation. Graphs show grand averages (coloured lines) ± SD (shaded area) of 19 participants. *C*, Latencies of LAS‐ and ES‐induced negative and positive response peaks in the left TA. Boxplots represent the median (black line) ± interquartile range (filled boxes) and mean values (black cross). Statistical differences are indicated by asterisks (^***^ *P* < 0.001).

During double support phase both coupling‐like and coupling responses were present in the left TA. A significant difference between LAS‐ and ES‐induced responses and baseline EMG activity was observed in the first time window (*P* < 0.001; Table 1). *Post hoc* analysis revealed that LAS, but not ES, evoked a negative response that differed significantly from baseline (*P* = 0.008; Table 4; Fig. 5*A*). However negative‐to‐positive peak latencies, characteristic of coupling and coupling‐like responses, were detected in 63.2% of all participants. Analogous to previous findings responses to ES were delayed compared to coupling‐like responses to LAS (N_CL_ *vs*. N_C_: T(11) = −6.122, *P* < 0.001, *r* = 0.172; P_CL_ *vs*. P_C_: T(11) = −4.642, *P* < 0.001, *r* = 0.206) (Table 5; Fig. 5*C*). In the right TA LAS‐ and ES‐induced responses differed significantly from baseline for both time windows (first time window: *P* < 0.001; second time window: *P* < 0.001; Table 1). *Post hoc* analysis showed that LAS and ES evoked positive responses that differed significantly from baseline (first time window LAS: *P* = 0.028; second time window LAS: *P* = 0.009; first time window ES: *P* < 0.001; second time window ES: *P* = 0.009; Table 4; Fig. 5*A*).

**Table 4:** Response size of the left and right tibialis anterior (TA) and gastrocnemius medialis (GM) during the double support in the walking task

Tibialis anterior				
	Baseline LAS	LAS	Effect size	*P*‐value (pairwise comparison)
***RMS_60‐100ms_ right [µV]***	**16.0 ± 6.3**	**25.3 ± 12.7**	**0.598**	0.028^*^
***RMS_100‐140ms_ right [µV]***	**16.2 ± 6.6**	**24.5 ± 10.7**	**0.821**	0.009^**^
***RMS_60‐100ms_ left [µV]***	**29.4 ± 11.9**	**24.2 ± 10.0**	**0.72**	0.008^**^
*RMS_100‐140ms_ left [µV]*	30.7 ± 12.0	33.0 ± 12.6	0.394	0.515

	Baseline ES	ES	Effect size	*P*‐value (pairwise comparison)
***RMS_90‐130ms_ right [µV]***	**16.1 ± 6.7**	**38.9 ± 28.0**	**0.914**	< 0.001^***^
***RMS_130‐170ms_ right [µV]***	**16.1 ± 5.6**	**29.5 ± 17.2**	**0.811**	0.009^**^
*RMS_90‐130ms_ left [µV]*	30.3 ± 12.0	28.8 ± 13.2	0.419	0.204
*RMS_130‐170ms_ left [µV]*	29.7 ± 11.1	32.2 ± 12.7	0.379	0.515

*Gastrocnemius medialis*				
	Baseline LAS	LAS	Effect size	*P*‐value (pairwise comparison)
*RMS_60‐100ms_ right [µV]*	41.5 ± 23.3	36.5 ± 18.0	0.157	0.834
*RMS_100‐140ms_ right [µV]*	53.0 ± 23.8	64.1 ± 40.9	0.479	0.186
*RMS_60‐100ms_ left [µV]*	7.7 ± 3.9	6.9 ± 3.0	0.538	0.11
*RMS_100‐140ms_ left [µV]*	8.0 ± 5.1	7.1 ± 2.4	0.043	1

	Baseline ES	ES	Effect size	*P*‐value (pairwise comparison)
*RMS_90‐130ms_ right [µV]*	49.8 ± 24.6	45.7 ± 27.3	0.389	0.359
*RMS_130‐170ms_ right [µV]*	63.1 ± 24.9	55.2 ± 24.2	0.419	0.272
*RMS_90‐130ms_ left [µV]*	7.6 ± 3.9	6.8 ± 3.2	0.072	1
*RMS_130‐170ms_ left [µV]*	9.4 ± 6.4	8.2 ± 3.9	0.043	1

*Note*: Loud acoustic stimuli (LAS)‐ and electrical stimuli (ES)‐induced responses were compared with baseline activity. Response component in the first time window is indicated by RMS_60–100ms_ for LAS‐induced responses and corresponding baseline activity, and by RMS_90–130ms_ for ES‐induced responses and corresponding baseline activity. Response component in the second time window is indicated by RMS_100–140ms_ for LAS‐induced responses and corresponding baseline activity, and by RMS_130–170ms_ for ES‐induced responses and corresponding baseline activity. Data represent mean values ± SD from 19 participants.

Statistical differences are indicated in bold letters and by asterisks (^*^ *P* < 0.05, **P < 0.01, ***P < 0.001).

**Table 5:** Latencies of coupling‐like and coupling responses

Ankle movement task ‐ left TA					
LAS		ES		Shift ES *vs*. LAS	
Latency [ms]		Latency [ms]		Latency [ms]	
	Mean ± SD		Mean ± SD	Mean ± SD	
N_CL_	78.3 ± 7.0	N_C_	111.4 ± 7.7	N_C_–N_CL_	33.1 ± 7.1
P_CL_	123.3 ± 12.61	P_C_	154.6 ± 16.3	P_C_–P_CL_	31.2 ± 26.4

Walking task – right swing phase ‐ left GM					
LAS		ES		Shift ES vs. LAS	
Latency [ms]		Latency [ms]		Latency [ms]	
	Mean ± SD		Mean ± SD	Mean ± SD	
N_CL_	88.3 ± 10.5	N_C_	121.0 ± 11.3	N_C_–N_CL_	32.7 ± 11.2
P_CL_	125.5 ± 10.5	P_C_	152.4 ± 17.2	P_C_–P_CL_	26.8 ± 16.1

Walking task – double support phase ‐ left TA					
LAS		ES		Shift ES *vs*. LAS	
Latency [ms]		Latency [ms]		Latency [ms]	
	Mean ± SD		Mean ± SD	Mean ± SD	
N_CL_	88.8± 12.2	N_C_	122.8 ± 12.9	N_C_–N_CL_	34.0 ± 19.2
P_CL_	129.5 ± 8.6	P_C_	151.8 ± 16.1	P_C_–P_CL_	22.6 ± 16.6

*Note*: Latencies of coupling‐like (LAS‐induced) and coupling responses (ES‐induced) in the left TA during the dorsiflexion task, the left GM during swing phase of the right leg and the left TA during the double support phase. Data represent mean values ± SD from 9 participants for the ankle task and 19 participants for the walking task.

Abbreviations: ES, electrical stimuli; GM, gastrocnemius medialis; LAS, loud acoustic stimuli; TA, tibialis anterior.

No coupling‐like and coupling responses appeared in the GM during double support. For the left GM no significant differences between LAS‐ and ES‐induced responses and baseline activity were identified (Table 1; Fig. 5*B*), and no negative‐to‐positive peak latencies were detected. Consequently no further analysis on coupling‐like and coupling responses was performed. In the right GM LAS and ES evoked responses that differed from baseline activity (first time window: *P* = 0.005; second time window: *P* = 0.01; Table 1). However pairwise comparisons showed no differences between LAS‐ and ES‐induced responses and baseline activity (Table 4; Fig. 5*B*).

### Configuration of coupling‐like and coupling responses

**Figure 6:**
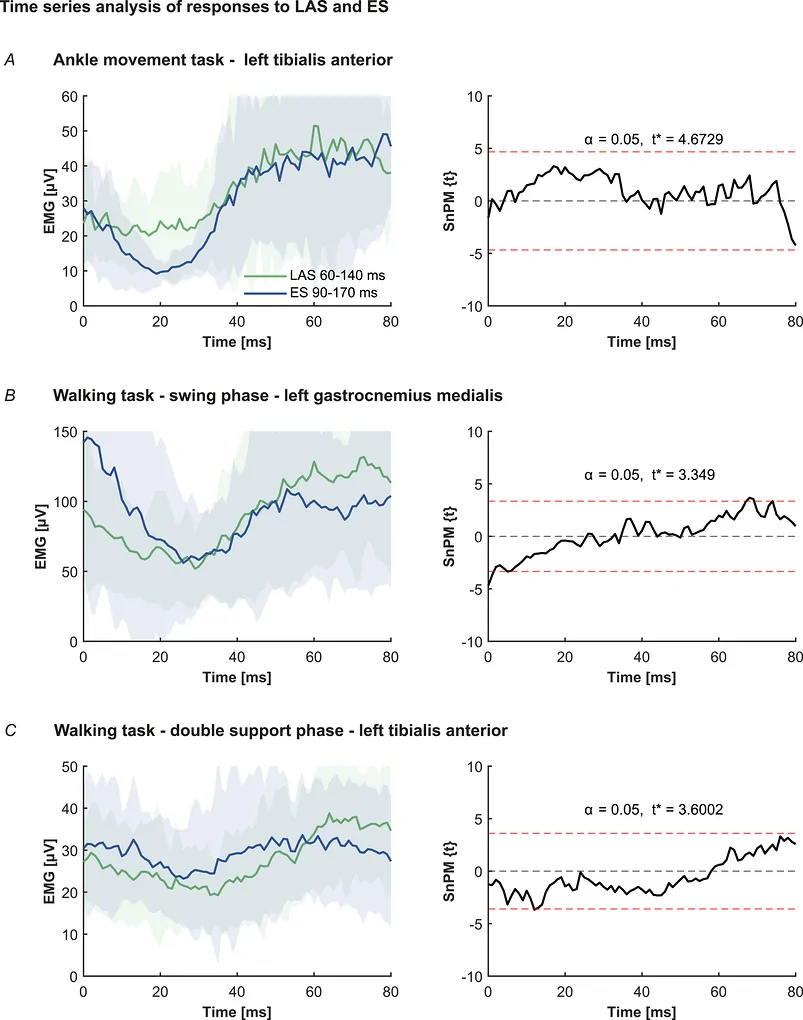
Time series analysis of EMG responses to loud acoustic stimuli (LAS) and electrical stimuli (ES) Left panels: overlay of averages of coupling‐like responses (LAS‐induced; green traces) from 60 to 140 ms after stimulus onset and of coupling responses (ES‐induced; blue traces) from 90 to 170 ms after stimulus onset: (*A*) left tibialis anterior (TA) responses during dorsiflexion task, (*B*) left gastrocnemius medialis (GM) responses during the right swing and, (*C*) left TA responses during the double support. Graphs show grand averages (coloured lines) ± SD (shaded area) of 19 participants. Right panels: results of SnPM {*t*} analysis comparing coupling‐like and coupling responses. The red dashed lines indicate the critical threshold *t* ^∗^ at α = 0.05. Any points or clusters that exceed this threshold are considered significantly different. SnPM, statistical non‐parametric mapping.

The identified coupling‐like and coupling responses to LAS and ES were compared across all muscles and tasks. A similarity of the coupling‐like and coupling responses was observed for both the ankle and the walking tasks. For the ankle task response modulations of coupling‐like and coupling response in the left TA were similar (first time window: *P* = 0.835, *r* = 0.270; second time window *P* = 1, *r* = 0.269). Time series analysis of the two responses (SnPM analysis) indicated no differences between coupling‐like and coupling responses (Fig. 6*A*). For the walking task coupling‐like and coupling responses of the left GM during the right swing showed no differences in response modulation (first time window: *P* = 0.108, *r* = 0.508; second time window: *P* = 0.136, *r* = 0.419). SnPM analysis of coupling‐like and coupling responses revealed that only a few data points at the beginning and at the end differed between LAS and ES evoked responses (t* > 3.349; Fig. 6*B*). During the double support phase response modulation did not differ between coupling‐like and coupling of the left TA (first time window: *P* = 0.149, *r* = 0.478; second time window: *P* = 0.723, *r* = 0.097). SnPM analysis confirmed these findings by showing that only a single data point was significantly different (t* > 3.600; Fig. 6*C*).

The time shift of ∼30 ms between the coupling‐like and the coupling response was consistent across muscles and tasks; that is the latency did not differ between the left TA during dorsiflexion (ankle task), the left GM during right swing and the left TA during double support (N_C_–N_CL_: H(2) = 0.107, *P* = 0.948, *F* = 0.232; P_C_–P_CL_: H (2) = 0.107, *P* = 0.948, *F* = 0.232; Table 5).

## Discussion

In this study a potential involvement of the reticulospinal system in the co‐ordination of leg movements during stepping was explored. EMG responses in the TA and GM muscles were analysed in response to ES of the right tibial nerve and to LAS during locomotion and, additionally, during voluntary ankle joint movements while sitting. Prominent EMG responses to both stimuli appeared during two assessed phases of gait, mainly in the left GM during the right swing and in the left TA during the double support. The responses to LAS and ES showed an analogous modulation across muscles and gait phases. Moreover a constant delay of 30 ms was observed between the responses to ES and LAS across all experimental conditions, aligning with their potential generation within brainstem circuits.

### Involvement of the brainstem in interlimb co‐ordination

Findings from preclinical experiments highlight the importance of the reticulospinal system for the co‐ordination of limb movements during locomotion, standing and reaching (Akalu et al., 2023). Little is known about the role of the reticulospinal system in the control of complex movements in humans, as tools to assess the function of subcortical motor systems are limited. A common approach to activate reticulospinal neurons in the pons and medulla is the application of LAS (Davis et al., 1982; Germann & Baker, 2021; Irvine et al., 1983). LAS can elicit auditory startle responses by activating reticulospinal neurons in the caudal pons (Yeomans & Frankland, 1996) but can also trigger other brainstem‐related responses, such as the StartReact effect (Tapia et al., 2022; Valls‐Solé et al., 1999) and auditory‐evoked vestibular responses (i.e. vestibular‐evoked myogenic potential (VEMP)) (Ashford et al., 2016; Dietz et al., 2024). Findings from these studies indicate that LAS‐induced coupling‐like responses are unlikely to reflect auditory startle responses.

In a recent study both ES and LAS were applied to explore a potential involvement of the reticulospinal system in the co‐ordination of hand movements. Indirect evidence suggests that the reticulospinal system contributes to the co‐ordination of bilateral hand movements by a ‘neural coupling’ (Dietz et al., 2024). This neural limb coupling is based on the observation that unilateral nerve stimulation results in EMG responses in limb muscles of both sides. In this study LAS elicited EMG responses that were similar in their configuration to those induced by ES. Moreover combined stimulation of LAS and ES led to a response pattern suggesting an interaction between the stimuli at the brainstem level (Dietz et al., 2024). Here a similar methodological paradigm (i.e. separate application of LAS and ES) was applied to investigate the contribution of the reticulospinal system to interlimb co‐ordination during stepping movements.

### Neural coupling during ankle joint movements

ES of the right tibialis nerve during voluntary ankle joint movements was followed by negative‐to‐positive responses in TA muscles of both sides, reflecting neural limb coupling. This finding is in line with a previous study showing bilateral reflex responses to unilateral nerve stimulation during bilateral limb movements (Balter & Zehr, 2007; Dietz et al., 2001). LAS evoked EMG responses that did not differ from those induced by ES. This corresponds to the findings made recently during bilateral hand movements (Dietz et al., 2024). Neural coupling responses to ES and LAS were only observed in the TA muscles during dorsiflexion movements but not in the GM, probably due to its low activation level. However given the relatively small sample size in the ankle movement tasks these findings should be interpreted cautiously.

The fact that also in the ankle task coupling responses occurred leads to the question of the limits of the coupling mechanism. It seems that neural coupling is generally present when limbs are moving and is even present in forearm muscles of a moving hand when the nerve of the relaxed contralateral arm was stimulated (Dietz et al., 2024). This may explain the difficulty in suppressing neural coupling during asynchronous hand movements requiring voluntary control (Köchli et al., 2020).

### Analogous modulation of responses during locomotion

During locomotion there was a similar and phase‐dependent response modulation to both LAS and ES across muscles. The responses were smallest in the GM during the double support phase of the step cycle. One might argue that in this phase the basic EMG activity was low, leading to small responses. However even in participants showing a stronger leg muscle activation response amplitudes were small or lacking. The largest responses appeared in the left GM, to both ES and LAS, during the right swing phase. During this phase of the gait cycle the left leg has to fulfil high balance requirements. Similarly responses to both stimuli were present in the left TA during the double support phase. This might be due to the subsequent initiation of the left swing. However the responses were smaller in amplitude and occurred in less participants compared to the left GM during the right swing phase. Overall a similar modulation and configuration of the responses to both ES and LAS were observed across all experimental conditions. The strong responses might reflect an enhanced supraspinal drive to adequately control these critical phases of the step cycle. The similar behaviour of the responses provides some indirect evidence that the same neural structure, possibly the brainstem, might be responsible for their generation. A corresponding step phase‐dependent response modulation to LAS and a possible involvement of underlying brainstem structures were described earlier (Nieuwenhuijzen et al., 2000). Therefore it seems that there is not only quadrupedal organization of bipedal gait (Dietz, 2002) but also lower limb co‐ordination during locomotion controlled by brainstem structures. This might include the integration of proprioceptive feedback control in the co‐ordination of leg muscles during stepping (Santuz et al., 2022).

Although startle responses were observed in the SCM muscles, no startles appeared in leg muscles to both LAS and ES, not even in the initial trials. This is in line with the low incidence of startles in lower limb muscles (Valls‐Solé et al., 2008).

### Timing of the responses compatible with a brainstem origin

A consistent difference in latency between the EMG responses to ES and LAS was observed in lower leg muscles as well as between the startle responses in the SCM. ES‐evoked responses were delayed by approximately 30 ms compared to those induced by LAS across all experimental conditions. A similar time shift was recently described for startle responses evoked through LAS and ES in the SCM (Daher et al., 2024). Additionally previous studies applying LAS and ES stimuli showed that ES and LAS simultaneously converge in the reticular formation if ES (to the biceps brachii) is applied 10 ms before the acoustic stimulus (Foysal et al., 2016; Germann & Baker, 2021). Given the fact that somatosensory‐evoked potentials elicited from the upper extremity are approximately 20 ms shorter than those from distal leg muscles a shift of 30 ms is consistent with an origin of the coupling response in the reticular formation. Thus it is argued that the longer latency of ES‐evoked responses might derive from differences in sensory conduction between the two stimuli. In fact somatosensory‐evoked cerebral potentials evoked by electrical tibial nerve stimulation appear with a latency of around 33 ms (N33). This is assumed to reflect brainstem activity below the thalamus (Urasaki et al., 1993). These are narrow normative values (mainly depending on the height of a subject) used in clinical electrophysiology. In contrast LAS‐induced activation of reticulospinal neurons takes place substantially faster, with a latency of around 7 ms in non‐human primates (Fisher et al., 2012) and humans (Foysal et al., 2016). Accordingly the difference in latency between ES‐ and LAS‐evoked responses in this study likely reflects different afferent conduction times and a time‐shifted arrival/integration of the two sensory modalities within brainstem structures. The efferent pathway to leg muscles seems to be identical for LAS‐ and ES‐evoked responses.

The present data provide indirect evidence that the neural limb coupling is mediated by a single neural structure (e.g. brainstem through spinal interneurons). This structure processes unilateral (peripheral or cortical) input and distributes the efferent output equally to the limbs of both sides. This ensures co‐ordination of limbs during complex movements. The contribution of other neural structures to the neural coupling mechanism appears rather unlikely.

### Functional consideration

The present findings provide indirect evidence that the reticulospinal system is involved in the control and co‐ordination of leg movements during locomotion, specifically during critical phases of the step cycle. The most prominent responses occurred in the left GM during the right swing, a gait phase where the body becomes ‘balanced’ over the left, body‐supporting leg. It is speculated that increased reticulospinal drive during this phase might be required to secure body equilibrium. A critical role of reticulospinal drive in balance control has been reported by several studies (Drew et al., 2004; Schepens et al., 2008; Takakusaki et al., 2017; Viseux et al., 2025). During double support marked responses appeared in the left TA muscle. This might reflect the critical phase of swing initiation where irregularities of the ground or obstacles should be avoided in preparation of the following step.

During the voluntary ankle task LAS and ES induced similar responses on both sides. In contrast the responses were differently modulated during walking on the right and left side, depending on the step cycle. Therefore reticulospinal drive might be regulated on supraspinal and/or spinal levels to provide functionally meaningful drive to the leg muscles of both sides. Here only two phases of the step cycle were investigated, that is the potential contribution of reticulospinal drive during other gait phases remains open.

## Conclusion

Little is known about the potential involvement of the reticulospinal system in the co‐ordination of human locomotion. Here LAS and ES were applied to explore the potential involvement of the reticulospinal system in interlimb co‐ordination during locomotion. Similarities in the behaviour and modulation of the responses to ES (reflecting neural limb coupling) and those to LAS (reflecting activation of the reticulospinal drive) imply a potential involvement of the brainstem, most probably the reticulospinal system, to neural lower limb co‐ordination during locomotion. These findings support the assumption that the reticulospinal system is involved not only in the co‐ordination of bilateral ankle joint movements but, likely, also in limb co‐ordination during locomotion.

## Additional information

## Competing interests

The authors declare no conflicts of interest.

## Author contributions

All experiments were performed at the Spinal Cord Injury Centre of the Balgrist University Hospital and the Swiss Centre for Movement Analysis, Balgrist Campus, Zurich, Switzerland. Conception or design of the work: N.S.H., V.D. and L.F. Acquisition of data: N.S.H., C.G. and M.G. Analysis of data: N.S.H. and C.G. Interpretation of data: N.S.H., V.D. and L.F. Drafting the work and revising it: N.S.H., V.D. and L.F. All authors have read and approved the final version of this manuscript and agreed to be accountable for all aspects of the work. All persons designated as authors qualify for authorship.

## Funding

The study was supported by the Swiss National Science Foundation (32003B_208110) and the Balgrist Foundation (2021‐079).

## Generative AI statement

No generative AI tools were used in the preparation of this manuscript.

## Supporting information


Peer Review History

## Data Availability

All data of this study are included in this manuscript (tables and figures). Raw data may be shared upon request.
