# Supplementary material for: Involvement of the reticulospinal system in the control of locomotion
Source: J Physiol. 2026 Sep 3;604(18):7869–89. doi: 10.1113/JP290941 (PMC13577682; doi:10.1113/JP290941)
Supplement: Supplementary file 1 [file TJP-604-7869-s001.pdf]

The Journal of Physiology

Involvement of the reticulospinal system in the control of locomotion

Nicole Sarah Holliger, Volker Dietz, Céline Grünenfelder, Marina Geissmann, and Linard Filli
DOI: 10.1113/JP290941

Corresponding author(s): Nicole Holliger (Nicole.Holliger@balgrist.ch)

The referees have opted to remain anonymous.

**Review Timeline:**		
	Submission Date:	15-Jan-2026
	Editorial Decision:	27-Apr-2026
	Revision Received:	24-Jun-2026
	Accepted:	10-Aug-2026

Senior Editor: Richard Carson

Reviewing Editor: Kathy Ruddy

Transaction Report:

(Note: With the exception of the correction of typographical or spelling errors that could be a source of ambiguity, letters and reports are not edited. Depending on transfer agreements, referee reports obtained elsewhere may or may not be included in this compilation. Referee reports are anonymous unless the Referee chooses to sign their reports.)

Re: JP-RP-2026-290941 "**Role of the brainstem in the control of locomotion**" by Nicole Sarah Holliger, Volker Dietz, Céline Grünenfelder, Marina Geissmann, and Linard Filli

Dear Dr Holliger,

Thank you for submitting your manuscript to The Journal of Physiology. It has been assessed by a Reviewing Editor and by 2 expert referees and we are pleased to tell you that it is potentially acceptable for publication following satisfactory major revision.

Please advise your co-authors of this decision as soon as possible.

The referee reports are copied at the end of this email.

Please address all the points raised and incorporate all requested revisions or explain in your Response to Referees why a change has not been made. We hope you will find the comments helpful and that you will be able to return your revised manuscript within 2 months. If your article is NOT for a Special Issue, you may have 9 months to revise. If you require an extension, please contact journal staff: jp@physoc.org. Please note that this letter does not constitute a guarantee for acceptance of your revised manuscript.

Your revised manuscript should be submitted online using the link in your Author Tasks: Link Not Available. This link is accessible via your account as Corresponding Author; it is not available to your co-authors. If this presents a problem, please contact journal staff (jp@physoc.org). Image files from the previous version are retained on the system. Please ensure you replace or remove any files that are being revised.

If you do not wish to submit a revised version of your manuscript, you must inform our journal staff (jp@physoc.org) or reply to this email to request withdrawal. Please note that a manuscript must be formally withdrawn from the peer review process at one journal before it may be submitted to another journal.

TRANSPARENT PEER REVIEW POLICY: To improve the transparency of its peer review process, The Journal of Physiology publishes online as supporting information the peer review history of all articles accepted for publication. Readers will have access to decision letters, including Editors' comments and referee reports, for each version of the manuscript, as well as any author responses to peer review comments. Referees can decide whether or not they wish to be named on the peer review history document.

ABSTRACT FIGURES: Authors are expected to use The Journal's premium BioRender account to create/redraw their Abstract Figures. Information on how to access this account is here: https://physoc.onlinelibrary.wiley.com/journal/14697793/biorender-access.

This will enable Authors to create and download high-resolution figures. If authors have used the free BioRender service, they can use the instructions provided in the link above to download a high-resolution version suitable for publication.

The link provided should only be used for the purposes of this submission. Authors will be charged for figures created on this account if they are not related to this manuscript submission.

LANGUAGE EDITING AND SUPPORT FOR PUBLICATION: If you would like help with English language editing, or other article preparation support, Wiley Editing Services offers expert help, including English Language Editing, as well as translation, manuscript formatting, and figure formatting at www.wileyauthors.com/eeo/preparation. You can also find resources for Preparing Your Article for general guidance about writing and preparing your manuscript at www.wileyauthors.com/eeo/prepresources.

RIGOUR AND REPRODUCIBILITY: We recommend authors consult The Journal's Rigour and Reproducibility requirements to ensure that their manuscript is suitable for publication: https://physoc.onlinelibrary.wiley.com/pb-assets/hub-assets/physoc/documents/TJP-Rigour-and-Reproducibility-Requirements.pdf

REVISION CHECKLIST:

Check that your Methods section conforms to journal policy: https://jp.msubmit.net/cgi-bin/main.plex?form_type=display_requirements#methods.

Check that data presented conforms to the statistics policy: https://jp.msubmit.net/cgi-bin/main.plex?form_type=display_requirements#statistics.

Upload a full Response to Referees file. To create your 'Response to Referees': copy all the reports, including any comments from the Senior and Reviewing Editors, into a Microsoft Word, or similar, file and respond to each point, using font or background colour to distinguish comments and responses and upload as the required file type.

Please upload two versions of your manuscript text: one with all relevant changes highlighted and one clean version with no changes tracked. The manuscript file should include all tables and figure legends, but each figure/graph should be uploaded as separate, high-resolution files.

You may also upload:

- 'Potential Cover Art' for consideration as the issue's cover image
- Appropriate Supporting Information (Video, audio or data set: see https://jp.msubmit.net/cgi-bin/main.plex?form_type=display_requirements#supp).

We look forward to receiving your revised submission.

If you have any queries, please reply to this email and we will be pleased to advise.

Yours sincerely,

Richard Carson
Senior Editor
The Journal of Physiology

REQUIRED ITEMS

- Author photo and profile. First or joint first authors are asked to provide a short biography (no more than 100 words for one author or 150 words in total for joint first authors) and a portrait photograph. These should be uploaded and clearly labelled together in a Word document with the revised version of the manuscript. See Information for Authors for further details.

- Papers must comply with the Statistics Policy: https://jp.msubmit.net/cgi-bin/main.plex?form_type=display_requirements#statistics.

In summary:

- If n {less than or equal to} 30, all data points must be plotted in the figure in a way that reveals their range and distribution. A bar graph with data points overlaid, a box and whisker plot or a violin plot (preferably with data points included) are acceptable formats.

- If n > 30, then the entire raw dataset must be made available either as supporting information, or hosted on a not-for-profit repository, e.g. FigShare, with access details provided in the manuscript.

- All relevant 'n' values must be clearly stated in the main text, figures and tables.

- Please include an Abstract Figure file and an Abstract Figure legend. An appropriate figure legend, which should not exceed 150 words in length, should be included in the main manuscript file. The Abstract Figure is a piece of artwork designed to give readers an immediate understanding of the research and should summarise the main conclusions. If possible, the image should be easily 'readable' from left to right or top to bottom. It should show the physiological relevance of the manuscript so readers can assess the importance and content of its findings. Abstract Figures should not merely recapitulate other figures in the manuscript. Please try to keep the diagram as simple as possible and without superfluous information that may distract from the main conclusion(s). Abstract Figures must be provided by authors no later than the revised manuscript stage and should be uploaded as a separate file during online submission labelled as File Type 'Abstract Figure'. Please also ensure that you include the figure legend in the main article file. All Abstract Figures should be created using BioRender. Authors should use The Journal's premium BioRender account to export high-resolution images. Details on how to use and access the premium account are included as part of this email.

EDITOR COMMENTS

Reviewing Editor:

While both referees were enthusiastic about the manuscript to some extent, they agree that there are fairly substantial changes required in order for the paper to be acceptable for The Journal of Physiology. Both feel that results are over-interpreted in some areas, and have concerns about the exclusion of many participants from some parts of the study. In

particular, Reviewer 2 believes that more experimental work is necessary to provide sufficient controls.

Senior Editor:

There are many commendable aspects of the submission that are recognised by the referees and editors. The study is well motivated, and in many respects well executed. It is also acknowledged that a very interesting set of observations has been described. There is however also consensus that the interpretations offered currently go well beyond those that can be supported by the current experimental methodology. While the pattern of outcomes may be consistent with the inferences made by the authors, they are evidently also compatible with other interpretations. There are two consequences that flow from this recognition. Firstly, as highlighted by Referee #2 in particular, additional control experiments will certainly be required, if the submission is to be given further consideration. In addition, as emphasised by both referees, a good deal more circumspection is required in presenting the results that have been obtained thus far. A further more general concern is that the final sample size entering some analyses is very small indeed. In such circumstances, it is difficult to have sufficient confidence that the effects reported for the sample are representative of those present in the population.

REFEREE COMMENTS

Referee #1:

This is a fantastic manuscript, and I commend all the authors for their effort. This study explores the brainstem's role in interlimb coordination during human walking. The authors employed unilateral tibial nerve electrical stimulation and loud acoustic stimulation during walking and ankle movements to try to identify a common neural basis for neural coupling across limbs. This approach is technically advanced and logically builds on previous work in this field. The authors demonstrate that LAS and ES-evoked muscle activity show similar patterns of modulation, and Leighton notes that this is intriguing and potentially useful. However, I have some concerns about the mechanistic claims and the underlying hypothesis, which require clarification and placement within a broader context. Additionally, improving overall internal consistency between the stated hypothesis, analysis, and conclusions would strengthen the manuscript. With appropriate revisions, this work could significantly contribute to the field.

The location of the reticulospinal pathway, as the shared neural pathway responsible for inter-limb coordination, is indirect and overstated in some places. Despite the convincing modulation and little evidence after muscle activity, none of these sources uniquely identify the reticulospinal tract. While LAS engages startle-related circuitry, it also activates auditory, vestibular, and other non-specific arousal systems. Also, the similarity of EMG waveforms does not uniquely imply a shared neural substrate underlying behavior. The authors should soften their language regarding the neural anatomical origin and adopt more cautious functional interpretations (i.e., consistent with reticulospinal drive...).

Many assumptions are made about unilateral tibial nerve stimulation as a specific, unified neural coupling mechanism, but isn't it also likely to involve multiple mechanisms and recruit a mix of afferent populations?

Could the authors clarify why the ES-evoked EMG activity across the limbs is thought to reflect a single coupling mechanism rather than a combination of spinal or supraspinal processes?

More context is needed to support the interpretation that constant latency differences between LAS- and ES-evoked responses suggest a shared neural source with varying afferent conduction times. Or, at the very least, soften language or claims that this localizes the brainstem. Other plausible mechanisms could also explain the near-constant offset.

Is it possible that LAS was consistently engaging the reticulospinal circuitry throughout the study? In this study, the SCM startle responses were rare and habituated across tasks. Further discussion of the relationship between the startle response and the EMG responses would benefit readers.

Was the EMG reported/quantified relative to a baseline/background?

The statistical analysis is sound. However, it could benefit from mapping each major analysis to a specific hypothesis. Additionally, it would be helpful to see effect sizes alongside p-values, especially considering the sample size and missing data (see below).

Some statements in the abstract, key points, and discussion go beyond what the data can support and suggest causality (e.g., mediated by the reticulospinal system).

The exclusion of half of the participants from the ankle task due to technical issues is significant and warrants a more detailed explanation.

Referee #2:

Role of the brainstem in the control of locomotion

This study addresses an important and timely question regarding the contribution of the reticulospinal system to interlimb coordination during locomotion. The experimental approach combining electrical stimulation (ES) and loud acoustic stimulation (LAS) is innovative, and the phase-specific analysis of gait is a clear strength. However, several conceptual and methodological issues limit the strength of the conclusions.

One aspect is that the central claim of a brainstem (reticulospinal) origin of the observed responses is not directly demonstrated but inferred. The interpretation of a constant latency shift (~30 ms) between LAS- and ES-induced responses as evidence for a shared brainstem generator is not compelling, as this difference can be explained by modality-specific afferent conduction times. Accordingly, statements suggesting that reticulospinal drive is "essential" for balance control are overstated and should be substantially toned down.

There are also conceptual inconsistencies regarding "neural coupling." Several times, ES is described as inducing neural coupling, whereas it should more accurately be considered a tool to probe or detect it. Moreover, the study applies a broadened concept of neural coupling across different tasks (walking and seated ankle movements) without clearly defining its underlying mechanisms or boundary conditions. Most critically, no true control condition is included in which bilateral (or even unilateral) limb activity occurs without neural coupling. As a result, it remains unclear whether the findings are specific to locomotion or reflect more general bilateral (or even unilateral) motor activity (see specific comments).

Methodologically, the absence of a combined LAS+ES condition is a missed opportunity, as such an approach could provide more direct evidence for interaction at the level of the reticulospinal system. The ankle task, presented as a control, does not fulfill this role since similar responses are observed. Additionally, the reduced sample size in this condition (n=9 due to exclusions) further weakens its interpretability.

In summary, while the study provides interesting observations on phase-dependent modulation of EMG responses after two different stimuli (LAS and ES), the conclusions regarding a specific role of the reticulospinal system in interlimb coordination during locomotion are not sufficiently supported by the data. A more cautious interpretation emphasizing that the findings are consistent with, but do not demonstrate, brainstem involvement would be more appropriate. Furthermore, the strong interpretations with regard to locomotion seem inappropriate as the same results were found in a non-locomotor task while the subjects were sitting.

Specific points:

Abstract

Line 31: "During stepping electrical stimuli (ES) were applied to the right tibial nerve to induce neural coupling of lower limbs, and loud acoustic stimuli (LAS) were released, known to activate the reticulospinal system."

- In the initial works (e.g. review Dietz 2002; Delwaide et al. 1984) it was correctly stated that the electrical stimuli were applied in order to test the neural coupling of the limbs. Here, the authors write the ES "induces" neural coupling. This is certainly not the case and should be rephrased as it may lead to serious misunderstandings.

Line 41: "These findings suggest that reticulospinal drive is dynamically modulated and that enhanced reticulospinal drive is essential to maintain body balance during locomotion."

- How did the authors demonstrate that "enhanced reticulospinal drive is essential to maintain body balance during locomotion"? This was not demonstrated in this study and should be removed.

Key points: "A constant time shift between responses to LAS and ES across all tasks indicates a common origin of their generation in the brainstem"

The title and the conclusion that it is the brainstem responsible for generating these responses is not directly measured but inferred. Too strong point is made.

Perhaps rather: Contribution of the reticulospinal tract in the control of locomotion

Introduction

- The introduction could be improved to better understand "neural coupling" as a neural mechanism for interlimb coordination that is only apparent when the limbs act together (e.g. gait or bilateral hand coordination). It is also important to better describe that this neural coupling can be tested: with electrical stimulation (neural coupling is NOT induced or provoked by ES!).

- It would also be good to describe the "evolution" with respect to the view on "neural coupling". In the initial works (e.g. Delwaide et al. 1984; referenced in Dietz review 2002), neural coupling occurred only in movements thought to involve central pattern generators such as gait BUT NOT during bilateral arm swing. Now, in this paper, neural coupling occurs in all kind of movements involving the left and right limbs, where we would not expect the central pattern generator playing a great role. Thus, the authors should elaborate a bit more on this wider view on "neural coupling". Also, having this much broader view on "neural coupling", it seems important to have control conditions, in which activity of left and right limbs are apparent but without neural coupling.

- Then, it is important for the introduction to provide the reader with the information that LAS is a well-established method to indicate the involvement of the reticulospinal tract. Thus, the LAS can be used to test the long-held assumption that neural coupling (and thus the responses after ES in coupled movements) are (mainly) determined by the brainstem. At the moment, the introduction lists a lot of previous works from the authors without making clear what is new and why the study at hand is important. A reason for this unclarity is that the first part of the introduction often implies that it was already shown that brainstem structures are important for locomotion. Thus, the reader who does not know what had actually been shown in previous human studies may have the impression that the study at hand (i.e. the experiment with LAS) is not necessary.

Methods

Line 127: "The experimental setup has been described in detail previously (Dietz et al., 2024)."

- This previous study investigated bilateral hand movements. It is therefore highly unlikely that this study described the experimental setup for the study at hand. Please delete this reference here and describe the experimental procedure in detail.

Line 129: "Unilateral ES were applied to the distal part of the right tibial nerve to elicit a neural 'coupling response', i.e. an EMG response not only in the ipsilateral, but also the contralateral (left) leg muscles (Balter & Zehr, 2007; Dietz, 2002)."

- This should be described better. In my understanding, neural coupling is only evident when the two limbs work together, originally thought to pinpoint to activity of a 'central pattern generator' (see Dietz 2002). Thus, we would expect neural coupling during the gait cycle but not during sitting or standing. This "neural coupling" framework is important to better understand the entire experiment. It is therefore advised to explain the "neural coupling" (also during bilateral hand coordination) and the potential involvement of brainstem structures (i.e. the reticulospinal formation) for this coupling in more detail. Please make sure that the ES is used to "test" or "detect" this neural coupling - not to induce neural coupling. Based on this, clear hypotheses should be stated when a) the brainstem is indeed responsible for this coupling or b) what is expected when the brainstem is not the relevant structure for this coupling. The involvement/non-involvement of the reticulospinal system would consequently influence the responses of the electrical stimulation and the LAS similarly/differently (in what way?)... Is this the only explanation or could we have a higher order control affecting both responses in a similar way? Or even a spinal mechanisms?

Line 161: Please cite "Zeni et al., (2008)" correctly.

General methodological questions:

- Why were ES and LAS not applied together? LAS should have an effect on the ES response (i.e. the coupling response) when this is propagated via the reticulospinal tract. This would be a more direct measure than the comparison across gait phases between the two kind of stimulations. If this was done and did not work, the authors should report this. If it was not done, it would be worth considering this for the revision - at least in a small sample size (with a control condition testing modulation of cortically evoked MEPs).

- Why was no control condition integrated? A task in which no neural coupling should be evident. Applying this rather broad concept of neural coupling in the present study makes it utterly important to have a control condition with activity in the left and right limb without neural coupling. Otherwise, everything in the paper has to be changed such as title, abstract, discussion, conclusion as the authors could not specifically say something about locomotion but rather about bilateral limb activity in general.

Discussion

Line 499: "EMG responses in the TA and GM muscles were analysed in response to ES of the right tibial nerve and to LAS during locomotion and, for control, during ankle joint movements."

- Here (and in introduction line 107), it is stated that EMG responses were analyzed during ankle joint movements as control. However, it is not clear what was expected in this control condition. When I had read the introduction for the first time, I had the impression this condition should be a "non-coupling" control condition. However, the results (and also the previous work in the upper-limbs) show differently. In what kind can the authors consider this as a control condition? There is similar neural coupling in this condition; and it is not a locomotion task. Based on this, the overall conclusion should rather not focus on locomotion but on bilateral limb movements.

Line 524: "Moreover, combined stimulation of LAS and ES led to a response pattern suggesting an interaction of the stimuli on a brainstem level (Dietz et al., 2024). Here, the same methodological paradigm (i.e. LAS and ES) was applied to investigate the contribution of the reticulospinal system to interlimb coordination during stepping movements."

- Can you really say that you have used the same methodological paradigm without combing the two stimuli? I think in this study there was no combination of LAS and ES (see my previous comments for the methods section)

Line 528 - 536: "Neural coupling during ankle joint movements"

- Based on the fact that no control condition was applied demonstrating the non-occurrence of "neural coupling", this statement is difficult to maintain. Perhaps every single limb movement results in "neural coupling"? It is perhaps rather a question of the threshold that has to be reached at the motor neuron pool. Thus, isometric activation of the left TA while the right TA makes a dorsiflexion movement might also cause a "coupling response" whereas this response might not be detectable when the left TA is at rest. (But not even the latter control condition (TA at rest) was integrated in the present study).

END OF COMMENTS

Point by point response

Date: 23 June 2026
Manuscript ID: JP-RP-2026-290941
Title of Article: Involvement of the reticulospinal system in the control of locomotion
Name of the Corresponding Author: Nicole Sarah Holliger
Email Address of the Corresponding Author: nicole.holliger@balgrist.ch

EDITOR COMMENTS

Reviewing Editor:

While both referees were enthusiastic about the manuscript to some extent, they agree that there are fairly substantial changes required in order for the paper to be acceptable for The Journal of Physiology. Both feel that results are over-interpreted in some areas, and have concerns about the exclusion of many participants from some parts of the study. In particular, Reviewer 2 believes that more experimental work is necessary to provide sufficient controls.

Senior Editor:

There are many commendable aspects of the submission that are recognised by the referees and editors. The study is well motivated, and in many respects well executed. It is also acknowledged that a very interesting set of observations has been described. There is however also consensus that the interpretations offered currently go well beyond those that can be supported by the current experimental methodology. While the pattern of outcomes may be consistent with the inferences made by the authors, they are evidently also compatible with other interpretations. There are two consequences that flow from this recognition. Firstly, as highlighted by Referee #2 in particular, additional control experiments will certainly be required, if the submission is to be given further consideration. In addition, as emphasised by both referees, a good deal more circumspection is required in presenting the results that have been obtained thus far. A further more general concern is that the final sample size entering some analyses is very small indeed. In such circumstances, it is difficult to have sufficient confidence that the effects reported for the sample are representative of those present in the population.

Response to the Editors

Dear Editors,

many thanks for your feedback with the decision that our manuscript is potentially acceptable for publication in The Journal of Physiology. We are grateful to the Editors and Referees for their very constructive and helpful comments. We were able to address most of the points raised by the editors and reviewers and are convinced that the quality of the revised manuscript has improved.

We find it, however, difficult to understand the need for further experiments on the basis of the comments made by reviewer 2. We have discussed and questioned this issue in the response to this reviewer.

The reviewer questions the lack of a proper control condition where a neural coupling response is absent. Indeed, we performed such a control experiment already in a previous study, where neural coupling was examined during unilateral hand movements, while the nerve of the relaxed forearm was stimulated. Even in such a condition, EMG responses were present in the forearm muscles of the moving hand (Dietz et al., 2024). We are not aware of a 'control condition' (tested in a number of upper limb movements) where neural coupling is absent. Today we know that all movement tasks so far studied show signs of neural coupling, except when limb muscles are relaxed (no EMG activity present).

In the present paper, we aimed to compare the neural coupling mechanisms between bilateral voluntary ankle movements, and more automatically performed stepping movements. The fact that neural coupling responses occurred in the ankle movement task is in line with our previous findings testing bilateral hand movements (Dietz et al., 2024).

A further request of this reviewer was to apply combined LAS/ES stimuli: In the present study, we planned NOT to repeat this kind of combined stimulation for two reasons: First, combined stimulations were already performed in the earlier study in order to assess the mechanistic interaction between LAS and ES study without providing extra insights in the neural coupling mechanism. In the current study, the focus was to investigate the potential involvement of the reticulospinal system in the more automated/complex task of walking.

Second, the present paper represents already a large scale study where two stimulation conditions in three different motor tasks were studied (while three stimulation conditions in only one motor task was examined in the earlier study (Dietz et al., 2024). An additional stimulation task in the present study would have needed about double time and expenditure for an experiment already performed in a previous study. Furthermore, such an expansion of the experiments would not have been reasonable as the increased number of applied stimuli could have been perceived as too intense and hardly tolerable for most volunteers.

On the basis of these arguments, we are convinced that this study already provides substantial new results for a sound interpretation and that it would not much profit from additional experiments.

Therefore, we would ask you to reconsider your decision and not to insist in the request to perform additional experiments.

Best regards,

the authors

<u>Responses to Reviewers:</u>
We would like to thank the reviewers for their helpful and constructive feedback on our manuscript. We have addressed all of comments raised in the point-by-point responses below. Based on the reviewer's comments, we have incorporated several relevant changes into the manuscript.

<u>Referee #1:</u>
This is a fantastic manuscript, and I commend all the authors for their effort. This study explores the brainstem's role in interlimb coordination during human walking. The authors employed unilateral tibial nerve electrical stimulation and loud acoustic stimulation during walking and ankle movements to try to identify a common neural basis for neural coupling across limbs. This approach is technically advanced and logically builds on previous work in this field. The authors demonstrate that LAS and ES-evoked muscle activity show similar patterns of modulation, and Leighton notes that this is intriguing and potentially useful. However, I have some concerns about the mechanistic claims and the underlying hypothesis, which require clarification and placement within a broader context. Additionally, improving overall internal consistency between the stated hypothesis, analysis, and conclusions would strengthen the manuscript. With appropriate revisions, this work could significantly contribute to the field.

<u>Response to Referee #1:</u>
Comment 1: *The location of the reticulospinal pathway, as the shared neural pathway responsible for inter-limb coordination, is indirect and overstated in some places. Despite the convincing modulation and little evidence after muscle activity, none of these sources uniquely identify the reticulospinal tract. While LAS engages startle-related circuitry, it also activates auditory, vestibular, and other non-specific arousal systems. Also, the similarity of EMG waveforms does not uniquely imply a shared neural substrate underlying behavior. The authors should soften their language regarding the neural anatomical origin and adopt more cautious functional interpretations (i.e., consistent with reticulospinal drive...).*

Response 1: According to the reviewers' comment, we adjusted strong statements concerning the involvement of the reticulospinal system in interlimb coordination in our manuscript to avoid overinterpretation. In fact, the evidence for an involvement of the reticulospinal system in neural coupling is indirect. This is now described more clearly in the revised discussion section. We are aware that LAS can activate not only the reticulospinal system but several neural structures such as the reticulo- and vestibulospinal systems (see also response 10 to reviewer 2). This was extensively described in our preceding paper where we characterized LAS-evoked responses (Dietz et al., 2024). In the present study, we have focused on the main responses (i.e., neural coupling responses, responses indicated by the negative-to-positive peaks) where we suggest that they are mediated by the reticulospinal system.
We now discuss the potential effects of LAS on different neural structures (i.e., vestibulospinal system) in more detail in the revised manuscript.

Comment 2: *Many assumptions are made about unilateral tibial nerve stimulation as a specific, unified neural coupling mechanism, but isn't it also likely to involve multiple mechanisms and recruit a mix of afferent populations?*
Could the authors clarify why the ES-evoked EMG activity across the limbs is thought to reflect a single coupling mechanism rather than a combination of spinal or supraspinal

processes?

Response 2: We agree that this question is essential and obviously was not yet convincingly described. Indeed, the unilateral afferent volley induced by the electrical stimulus consists in a mix of afferent fibres. However, the EMG responses evoked were always the same on the stimulated AND the non-stimulated side. As shown in an earlier experiment (cf fig. 3 in Thomas et al., 2018), during an 'opening a bottle'-motor task, background EMG activity increases on both sides with the force exerted and the movement velocity. The reflex amplitudes following unilateral nerve stimulation showed a linear increase with this level of EMG activity, i.e. showed an automatic reflex gain compensation on the stimulated AND the non-stimulated side. This observation is hardly compatible with the view of shared spinal and supraspinal contributions to the responses.
Accordingly, the neural coupling mechanism apparently consists of a single neural structure (e.g. brainstem through spinal interneuronal circuits that processes (unilateral peripheral or cortical input) and distributes the efferent output equally to both sides (upper or /and lower limb muscles) coordinating limb movements. A contribution of different neural structures appears rather unlikely.
In the revisions we have better explained the arguments for a single neural structure responsible for neural limb coupling.

Comment 3: *More context is needed to support the interpretation that constant latency differences between LAS- and ES-evoked responses suggest a shared neural source with varying afferent conduction times. Or, at the very least, soften language or claims that this localizes the brainstem. Other plausible mechanisms could also explain the near-constant offset.*

Response 3: We agree with the notion to be more cautious with our claims and have made appropriate corrections. Our latency suggestions are to a large part based on the quite narrow normative values of latencies from somatosensory evoked potentials (SSEP) in clinical settings (range depends mainly on body height). These values for brainstem vs. cortex correspond to those described in our study (Urasaki et al., 1993). Moreover, in an earlier paper, combined application of LAS and ES showed that ES and LAS simultaneously converge in the reticular formation, if ES (to the biceps brachii) is applied 10 ms before the acoustic stimulus (Germann et al., 2021). Given that SSEPs elicited from the upper extremity are approximately 20 ms shorter than those from distal lower limb muscles, this further supports the assumption that a shift of 30 ms aligns with a suggested origin of the coupling response in the reticular formation. Please also see response 1 to Reviewer 2. In the revisions we have addressed this point more clearly and were more cautious in the interpretation of this latency shift**.**

Comment 4: *Is it possible that LAS was consistently engaging the reticulospinal circuitry throughout the study? In this study, the SCM startle responses were rare and habituated across tasks. Further discussion of the relationship between the startle response and the EMG responses would benefit readers.*

Response 4: LAS can elicit several distinct brainstem-mediated motor responses, including the auditory startle response, StartReact-associated responses, and — as hypothesized in the present study — the coupling-like response. In our previous work (Dietz et al., 2024), we identified clear differences between the acoustic startle response and the coupling-like

response. Startle responses occurred during both resting and movement conditions, whereas coupling-like responses were observed exclusively during movement. In addition, startle responses exhibited distinct configurations and latencies compared with the coupling-like responses. Furthermore, startle responses exhibit a characteristic habituation, reported in earlier studies (Ornitz & Guthrie, 1989; Valls-Solé et al., 2008), whereas coupling-like responses showed no evidence for habituation (this was also observed in the present study). Finally, we observed a strong interaction between startle and coupling-like responses: the onset of the coupling-like response was delayed by the duration of the startle response. Together, these findings support the interpretation that the startle and coupling-like responses represent two distinct responses. Given the absence of habituation in the LAS- and ES-induced coupling responses, we are convinced that LAS induce an activation of reticulospinal neurons throughout the experimental session.

Comment 5: *Was the EMG reported/quantified relative to a baseline/background?*

Response 5: The EMG response modulation after ES and LAS were compared to the baseline activity by the root mean squares (RMS), (see tables 2,3, and 4 in our manuscript). We did not perform any normalization of the evoked responses because we were interested in the detailed timing and absolute amplitudes of the EMG response modulation. All measurements were performed within the same session (i.e., with the same electrode placement and overall setup), allowing to accurately compare EMG responses across conditions without further normalization.

Comment 6: *The statistical analysis is sound. However, it could benefit from mapping each major analysis to a specific hypothesis. Additionally, it would be helpful to see effect sizes alongside p-values, especially considering the sample size and missing data (see below).*

Response 6: In the “Results” section, we have now specified the hypothesis and the corresponding statistical analyses more clearly. Effect sizes for the various analyses were not specified in the manuscript text but are reported in the tables referred before reporting the p-values. Detailed statistical outcomes can be found in these tables.

Comment 7: *Some statements in the abstract, key points, and discussion go beyond what the data can support and suggest causality (e.g., mediated by the reticulospinal system).*

Response 7: We now avoid strong statements in the abstract, key points, and discussion. The key point described has been changed to: “Indirect evidence suggests that neural leg coupling during stepping is mediated by brainstem structures, likely the reticulospinal system”

Comment 8: *The exclusion of half of the participants from the ankle task due to technical issues is significant and warrants a more detailed explanation.*

Response 8: Ten participants were excluded from the ankle task due to a deficient stimulus triggering mechanism regarding both LAS and ES. This resulted in stimuli being released at random time points that did not coincide with the planned ones. Unfortunately, this malfunction was only detected and resolved after ten participants. Deficient stimulus triggering only occurred in the setup of the ankle task, but not during locomotor conditions.

Referee #2:

Role of the brainstem in the control of locomotion
This study addresses an important and timely question regarding the contribution of the reticulospinal system to interlimb coordination during locomotion. The experimental approach combining electrical stimulation (ES) and loud acoustic stimulation (LAS) is innovative, and the phase-specific analysis of gait is a clear strength. However, several conceptual and methodological issues limit the strength of the conclusions.

Response to Referee #2:
Comment 1: *One aspect is that the central claim of a brainstem (reticulospinal) origin of the observed responses is not directly demonstrated but inferred. The interpretation of a constant latency shift (~30 ms) between LAS- and ES-induced responses as evidence for a shared brainstem generator is not compelling, as this difference can be explained by modality-specific afferent conduction times. Accordingly, statements suggesting that reticulospinal drive is "essential" for balance control are overstated and should be substantially toned down.*

Response 1: In fact, we hypothesized that the observed shift of ~30 ms reflects modality-specific differences in afferent conduction times. This is supported by electrophysiological standard values from tibialis SSEPs (Urasaki et al., 1993) as well as acoustically and electrically induced startle responses (Daher et al., 2024), as mentioned in the manuscript. Moreover, a study applying combined LAS and ES stimuli showed that ES and LAS simultaneously converge in the reticular formation, if ES (to the biceps brachii) is applied 10 ms before the acoustic stimulus (Germann et al., 2021). Given the fact that SSEPs elicited from the upper extremity are approximately 20 ms shorter than those from distal lower extremity muscles, a shift of 30 ms aligns with a potential origin of the coupling response in the reticular formation. However, we interpreted the latency shift more carefully in the revised manuscript.
We agree and toned down our overstated suggestions concerning control of balance. Nevertheless, several previous studies claimed that the reticulospinal system plays an important role in maintaining balance during various postural tasks and locomotion (Viseux et al., 2025, Drew et al., 2004, Takakusaki et al., 2017, Schepens et al., 2008). To maintain balance, the reticulospinal system interacts with various inputs from supraspinal, spinal structures and peripheral receptors. As the reticulospinal system is not the only system involved in balance control, we adapted our statements concerning balance control in the discussion. We have removed the statement in the abstract regarding the role of the reticulospinal system in balance control.

Comment 2: *There are also conceptual inconsistencies regarding "neural coupling." Several times, ES is described as inducing neural coupling, whereas it should more accurately be considered a tool to probe or detect it. Moreover, the study applies a broadened concept of neural coupling across different tasks (walking and seated ankle movements) without clearly defining its underlying mechanisms or boundary conditions. Most critically, no true control condition is included in which bilateral (or even unilateral) limb activity occurs without neural coupling. As a result, it remains unclear whether the findings are specific to locomotion or reflect more general bilateral (or even unilateral) motor activity (see specific comments).*

Response 2: This indeed represents an important conceptual point. First: In fact, the reviewer is right that the 'neural coupling' evoked by ES reflects/probes a limb coordination mechanism. Second, the following point concerns the 'ankle task' as 'control' condition: One aim of the study was to investigate the neural coupling mechanism in two fundamentally different motor tasks, i.e., in a voluntarily conducted task (ankle task), and a complex, more automated task (locomotion). We now describe this more clearly in the manuscript. Third, up to now, we do not know any motor task without neural coupling. Even nerve stimulation of a relaxed arm showed a response in the contralateral moving arm (Dietz et al., 2024). It is therefore rather unlikely that a control task exists where NO neural coupling takes place (see also response 15).
In the revisions, we have more extensively explained the different aspects of the neural coupling mechanism.

Comment 3: *Methodologically, the absence of a combined LAS+ES condition is a missed opportunity, as such an approach could provide more direct evidence for interaction at the level of the reticulospinal system. The ankle task, presented as a control, does not fulfill this role since similar responses are observed. Additionally, the reduced sample size in this condition (n=9 due to exclusions) further weakens its interpretability.*

Response 3: The focus of this study was to investigate the coupling and coupling-like response during separately applied ES and LAS across various tasks (i.e., ankle task, swing phase and double support phase during walking task) and muscles (TA and GM). However, it was not the aim to further investigate the neural substrates underlying neural coupling. This was the focus of our previous study (Dietz et al., 2024). Therefore, we decided not to perform combined stimulation in the current study, as we did not expect further support for our hypothesis. Please also see response 14 for a more detailed explanation of why we did not implement the combined LAS and ES stimulation in this study.
The voluntary ankle task was planned as an alternative motor condition for the more complex and automatically performed walking task (transition from the bilateral hand task to walking). The ankle task performed while sitting provided information about a potential neural coupling in such an alternative task. The term “control task” was used to refer to a voluntarily performed control task, in contrast to the automatically performed walking. We revised the term “control task” in our manuscript in the “Introduction” and “Discussion” sections.
We are aware of the limitations due to the small sample size in the ankle task and have added this as a limitation to our manuscript.

Comment 4: *In summary, while the study provides interesting observations on phase-dependent modulation of EMG responses after two different stimuli (LAS and ES), the conclusions regarding a specific role of the reticulospinal system in interlimb coordination during locomotion are not sufficiently supported by the data. A more cautious interpretation emphasizing that the findings are consistent with, but do not demonstrate, brainstem involvement would be more appropriate. Furthermore, the strong interpretations with regard to locomotion seem inappropriate as the same results were found in a non-locomotor task while the subjects were sitting.*

Response 4: As requested by the reviewer, we adjusted our “Discussion” section regarding the role of the reticulospinal system in locomotor coordination and avoided too strong interpretations.

The coupling responses during the ankle task align with our previous observations during a bilateral hand movement task (Dietz et al., 2024). These findings show that neural coupling is also present in non-locomotor tasks that do not primarily rely on CPG networks. However, we observed distinct differences in neural coupling between tasks: While the strongest neural coupling responses during the ankle task appeared in the TA, the strongest neural coupling response during locomotion was present during the right swing phase in the left GM. During the double support phase neural coupling responses appeared in the left TA. These responses were smaller in amplitude and appeared in less participants (lower incidence) compared to the neural coupling of TA in the ankle task. These observations highlight important differences in neural coupling responses between the motor tasks. We discuss these aspects more specifically in the revised "Discussion" section.

Comment 5: *Specific points: Abstract. Line 31: "During stepping electrical stimuli (ES) were applied to the right tibial nerve to induce neural coupling of lower limbs, and loud acoustic stimuli (LAS) were released, known to activate the reticulospinal system."*
In the initial works (e.g. review Dietz 2002; Delwaide et al. 1984) it was correctly stated that the electrical stimuli were applied in order to test the neural coupling of the limbs. Here, the authors write the ES "induces" neural coupling. This is certainly not the case and should be rephrased as it may lead to serious misunderstandings.

Response 5: The reviewer is right. The bilateral responses to unilateral nerve stimulation probe the 'neural coupling' mechanism.
This is appropriately corrected in the revised version of the manuscript.

Comment 6: *Line 41: "These findings suggest that reticulospinal drive is dynamically modulated and that enhanced reticulospinal drive is essential to maintain body balance during locomotion."*
How did the authors demonstrate that "enhanced reticulospinal drive is essential to maintain body balance during locomotion"? This was not demonstrated in this study and should be removed.

Response 6: We removed the mentioned statement from the abstract. We now discuss and speculate on the dynamic influence of the reticulospinal system on balance control in the revised discussion section. In fact, the single leg stance phase represents a critical phase of the step cycle with high balance demands. The strong reticulospinal drive in the left GM during this phase of the step cycle might be associated with the requirement of an enhanced reticulospinal drive in this phase of the step cycle.

Comment 7: *Key points: "A constant time shift between responses to LAS and ES across all tasks indicates a common origin of their generation in the brainstem"*
The title and the conclusion that it is the brainstem responsible for generating these responses is not directly measured but inferred. Too strong point is made.
Perhaps rather: Contribution of the reticulospinal tract in the control of locomotion

Response 7: We changed the key point statement to the following: "A 30 ms time shift between responses to LAS and ES across all tasks is compatible with a common origin of their generation in the brainstem". Regarding the interpretation of the time shift between LAS- and ES-evoked responses, please also see response 1.

We adjusted our title to: “Involvement of the reticulospinal system in the control of locomotion”

Comment 8: *Introduction. The introduction could be improved to better understand "neural coupling" as a neural mechanism for interlimb coordination that is only apparent when the limbs act together (e.g. gait or bilateral hand coordination). It is also important to better describe that this neural coupling can be tested: with electrical stimulation (neural coupling is NOT induced or provoked by ES!).*

Response 8: We agree and have implemented the expression proposed by the reviewer: 'neural coupling’ as a neural mechanism for interlimb coordination that is usually apparent when the limbs act together (e.g. gait or bilateral hand coordination). Furthermore: ES-evoked responses reflect/test the neural limb coupling mechanism.

Comment 9: *It would also be good to describe the "evolution" with respect to the view on "neural coupling". In the initial works (e.g. Delwaide et al. 1984; referenced in Dietz review 2002), neural coupling occurred only in movements thought to involve central pattern generators such as gait BUT NOT during bilateral arm swing. Now, in this paper, neural coupling occurs in all kind of movements involving the left and right limbs, where we would not expect the central pattern generator playing a great role. Thus, the authors should elaborate a bit more on this wider view on "neural coupling". Also, having this much broader view on "neural coupling", it seems important to have control conditions, in which activity of left and right limbs are apparent but without neural coupling.*

Response 9: We, to a large part, agree with this comment and have expanded this section in the revised version.
Indeed, neural limb coupling occurs in a wide range of movements. So far tested, neural coupling is even apparent during single hand movements when the nerve of the relaxed side is stimulated (cf Dietz et al. 2024). We would suggest that the neural limb coupling mechanism is strongly linked with the CPG during locomotion, including arm swing, that reflects quadrupedal organization of bipedal gait (Dietz 2002). However, neural coupling is also evident in limb movements where CPG networks are not involved. Please see also response 15 below.

Comment 10: *Then, it is important for the introduction to provide the reader with the information that LAS is a well-established method to indicate the involvement of the reticulospinal tract. Thus, the LAS can be used to test the long-held assumption that neural coupling (and thus the responses after ES in coupled movements) are (mainly) determined by the brainstem. At the moment, the introduction lists a lot of previous works from the authors without making clear what is new and why the study at hand is important. A reason for this unclarity is that the first part of the introduction often implies that it was already shown that brainstem structures are important for locomotion. Thus, the reader who does not know what had actually been shown in previous human studies may have the impression that the study at hand (i.e. the experiment with LAS) is not necessary.*

Response 10: We now expanded the section in the introduction on the use of LAS to probe the involvement of the reticulospinal system in movement control. We now mention that LAS activate reticulospinal neurons (Fisher et al., 2012), while neurons in the motor cortex are suppressed (Furubayashi et al., 2000; Tapia et al., 2022). LAS can elicit brainstem-related

related motor response including vestibular-evoked potentials and the auditory startle response (Brown et al., 1991), but also other responses including those associated with the StartReact effect (Tapia et al., 2022; Neumann et al., 2025). Additionally, we refined the section on the knowledge of reticulospinal involvement in locomotor control and now mention that most studies investigating reticulospinal contributions to locomotion were performed in animal models. In humans, the present study can contribute to the role of supraspinal motor centres, including the reticulospinal system, on locomotion remains partially open.

Comment 11: *Methods. Line 127: "The experimental setup has been described in detail previously (Dietz et al., 2024)." This previous study investigated bilateral hand movements. It is therefore highly unlikely that this study described the experimental setup for the study at hand. Please delete this reference here and describe the experimental procedure in detail.*

Response 11: We deleted this sentence, as we explained the experimental setup in detail in the following sections “Ankle task” and “Walking task”. We initially included this sentence to make it clear to readers that a similar protocol has been used in a previous study.

Comment 12: *Line 129: "Unilateral ES were applied to the distal part of the right tibial nerve to elicit a neural 'coupling response', i.e. an EMG response not only in the ipsilateral, but also the contralateral (left) leg muscles (Balter & Zehr, 2007; Dietz, 2002)."*
This should be described better. In my understanding, neural coupling is only evident when the two limbs work together, originally thought to pinpoint to activity of a 'central pattern generator' (see Dietz 2002). Thus, we would expect neural coupling during the gait cycle but not during sitting or standing. This "neural coupling" framework is important to better understand the entire experiment. It is therefore advised to explain the "neural coupling" (also during bilateral hand coordination) and the potential involvement of brainstem structures (i.e. the reticulospinal formation) for this coupling in more detail. Please make sure that the ES is used to "test" or "detect" this neural coupling - not to induce neural coupling. Based on this, clear hypotheses should be stated when a) the brainstem is indeed responsible for this coupling or b) what is expected when the brainstem is not the relevant structure for this coupling. The involvement/non-involvement of the reticulospinal system would consequently influence the responses of the electrical stimulation and the LAS similarly/differently (in what way?)... Is this the only explanation or could we have a higher order control affecting both responses in a similar way? Or even a spinal mechanisms?

Response 12: We have corrected this sentence as proposed: (thanks to the reviewer!).
As mentioned previously, neural coupling is evident in many motor tasks, including those that likely do not rely on CPG networks (see also responses 9 and 15). In all these movement tasks, ES and LAS evoked the same pattern of EMG responses in limb muscles involved in the respective motor task. According to recent studies, evidence exists that LAS activates the reticulospinal system (Fisher et al., 2012; see response 10, above). These observations provide indirect evidence that the reticulospinal system is involved in the neural coupling mechanism coordinating limbs during complex movements. Further, the latency shift between ES- and LAS-evoked responses are in line with the assumption that the neural coupling responses are mediated by the reticulospinal system (see also response 1).
As outlined above, the earlier observation of an automatic reflex compensation that depends on the level of background EMG, that exists not only on the stimulated but also on the non-stimulated side (cf fig.3 in Thomas et al. 2018) makes a shared (e.g. spinal or supraspinal)

contribution to the responses rather unlikely. Of course, these results/arguments reflect only indirect evidence.
We explain now the "neural coupling" and the potential involvement of brainstem structures (i.e. the reticulospinal formation) for this coupling independent of the CPG in more detail.

Comment 13: *Line 161: Please cite "Zeni et al., (2008)" correctly.*

Response 13: We now cite Zeni et al., 2008 correctly.

Comment 14: *General methodological questions: Why were ES and LAS not applied together? LAS should have an effect on the ES response (i.e. the coupling response) when this is propagated via the reticulospinal tract. This would be a more direct measure than the comparison across gait phases between the two kind of stimulations. If this was done and did not work, the authors should report this. If it was not done, it would be worth considering this for the revision - at least in a small sample size (with a control condition testing modulation of cortically evoked MEPs).*

Response 14: The neural substrate underlying the coupling response was the primary focus of our previous work (Dietz et al., 2024). Several observations provided supporting, indirect evidence for an involvement of the reticulospinal system in the neural coupling mechanism, including results obtained from the combined application of ES and LAS. The primary objective of the present study was to examine neural coupling across different muscles and during different phases of a more complex motor task (i.e., locomotion).

Comment 15: *Why was no control condition integrated? A task in which no neural coupling should be evident. Applying this rather broad concept of neural coupling in the present study makes it utterly important to have a control condition with activity in the left and right limb without neural coupling. Otherwise, everything in the paper has to be changed such as title, abstract, discussion, conclusion as the authors could not specifically say something about locomotion but rather about bilateral limb activity in general.*

Response 15: A control experiment investigating a motor condition where neural coupling responses are absent was conducted in our previous study (Dietz et al., 2024). In that work, neural coupling occurred even during unilateral hand movements, when the resting arm was stimulated (see Figure 5). Again, the configuration of the ES-evoked response was similar to the one evoked by LAS in the forearm extensors of the moving hand. In contrast, neither ES nor LAS produced responses in the extensor muscles of the relaxed arm, likely because the reflex amplitude did not exceed motor threshold. These observations suggest that neural coupling represents a robust mechanism. This may account for the difficulty to suppress neural coupling during asynchronous hand movements that require voluntary control (Köchli et al. 2020). Based on this evidence, additional control experiments in this direction would not substantially add to the findings of the present study. Instead, we have expanded the discussion to emphasize that neural coupling is not restricted to movements mediated by central pattern generator (CPG) networks but is evident in the performance of a wide range of everyday simple and complex movements.

Comment 16: *Discussion. Line 499: "EMG responses in the TA and GM muscles were analysed in response to ES of the right tibial nerve and to LAS during locomotion and, for control, during ankle joint movements."*

Here (and in introduction line 107), it is stated that EMG responses were analyzed during ankle joint movements as control. However, it is not clear what was expected in this control condition. When I had read the introduction for the first time, I had the impression this condition should be a "non-coupling" control condition. However, the results (and also the previous work in the upper-limbs) show differently. In what kind can the authors consider this as a control condition? There is similar neural coupling in this condition; and it is not a locomotion task. Based on this, the overall conclusion should rather not focus on locomotion but on bilateral limb movements.

Response 16: In our study, we planned bilateral ankle joint movements as 'control condition' to the stepping movements. Specifically, we examined the neural coupling mechanisms during two different motor tasks (i.e., a voluntary, non-locomotor task vs. a more automatically performed, locomotor task). The measurements resulted in the observation that, also in the 'ankle task', signs of neural coupling were present. In fact, the neural coupling responses in the ‘walking task’ were, in contrast to the ankle task, modulated differently in the two legs and the two phases of locomotion. Up to now, in all motor task so far studied, a neural coupling was present, i.e. a real 'control' condition without neural coupling was not yet detected.
In the revised version, we removed the term "control task" and explain the aim of the study, i.e. the neural coupling mechanisms in two different motor tasks.

Comment 17: *Line 524: "Moreover, combined stimulation of LAS and ES led to a response pattern suggesting an interaction of the stimuli on a brainstem level (Dietz et al., 2024). Here, the same methodological paradigm (i.e. LAS and ES) was applied to investigate the contribution of the reticulospinal system to interlimb coordination during stepping movements."*
Can you really say that you have used the same methodological paradigm without combing the two stimuli? I think in this study there was no combination of LAS and ES (see my previous comments for the methods section)

Response 17: Indeed, we did not apply the combination of LAS and ES in this study. We corrected the sentence in question as follows: “Here, a similar methodological paradigm (i.e. separate application LAS and ES) was used to investigate the contribution of the reticulospinal system to interlimb coordination during stepping movements."

Comment 18: *Line 528 - 536: "Neural coupling during ankle joint movements"*
Based on the fact that no control condition was applied demonstrating the non-occurrence of "neural coupling", this statement is difficult to maintain. Perhaps every single limb movement results in "neural coupling"? It is perhaps rather a question of the threshold that has to be reached at the motor neuron pool. Thus, isometric activation of the left TA while the right TA makes a dorsiflexion movement might also cause a "coupling response" whereas this response might not be detectable when the left TA is at rest. (But not even the latter control condition (TA at rest) was integrated in the present study).

Response 18: Yes, the reviewer is absolutely right! The neural coupling responses obtained during the ankle task correspond to those of the bilateral hand movement task described earlier (Dietz et al., 2024). In the latter study, the suggestion made by the reviewer could be verified: During unilateral hand movements, electrical nerve stimulation of the relaxed arm

resulted in an EMG response in the contralateral moving arm. In this study, no EMG responses were detected in the TA at rest.

Dear Dr Holliger,

Re: JP-RP-2026-290941R1 "Involvement of the reticulospinal system in the control of locomotion" by Nicole Sarah Holliger, Volker Dietz, Céline Grünenfelder, Marina Geissmann, and Linard Filli

We are pleased to tell you that your paper has been accepted for publication in The Journal of Physiology.
- Authors are required to fully disclose the use of any Generative AI (GAI) tools used in the preparation of their manuscript. This disclosure should also be included as a statement alongside the Additional Information and Data Availability statements towards the end of the article under the heading 'Generative AI Statement', and include the name(s), model and version of AI technology used, and exact details on the purpose for, and methods of, its use.

If no AI tool has been used, this should also be specifically stated within the manuscript.

The last Word (or similar) version of the manuscript provided will be used by the Production Editor to prepare your proof. When this is ready you will receive an email containing a link to Wiley's Online Proofing System. The proof should be thoroughly checked and corrected as promptly as possible.

Authors should note that it is too late at this point to offer corrections prior to proofreading. Major corrections at proof stage, such as changes to figures, will be referred to the Editors for approval before they can be incorporated. Only minor changes, such as to style and consistency, should be made at proof stage. Changes that need to be made after proof stage will usually require a formal correction notice.

All queries at proof stage should be sent to: TJP@wiley.com.

Following copyediting and typesetting the accepted version will be published online.

Yours sincerely,

Richard Carson
Senior Editor
The Journal of Physiology

IMPORTANT POINTS TO NOTE FOLLOWING ACCEPTANCE OF YOUR PAPER:

• IMPORTANT NOTICE ABOUT OPEN ACCESS: To assist authors whose funding agencies mandate immediate public access to published research findings, The Journal of Physiology allows authors to pay an Open Access (OA) fee to have their papers made freely available immediately on publication.

The Corresponding Author will receive an email from Wiley with details on how to register or log in to Wiley Authors where you will be able to place an order.

• You can check if your funder or institution has a Wiley Open Access Account here:
https://authors.wiley.com/author-resources/Journal-Authors/open-access/author-compliance-tool.html

MEMBER DISCOUNT: Corresponding authors who are Members of the Physiological Society are eligible for a 20% discount on the cost of the Article Processing Charge (APC). To claim this discount, the Corresponding Author will need to log in to the Physiological Society Membership portal and retrieve the discount code (https://www.physoc.org/journals-and-media/journals/journal-discount-for-members/). This code should then be quoted in the billing section of Wiley Authors when selecting the open access option.

• TRANSPARENT PEER REVIEW POLICY: To improve the transparency of its peer review process, The Journal of Physiology publishes online as supporting information the peer review history of all articles accepted for publication. Readers will have access to decision letters, including Editors' comments and referee reports, for each version of the manuscript as well as any author responses to peer review comments. Referees can decide whether or not they wish to be named on the peer review history document.

• You can help your research get the attention it deserves! Check out Wiley's free Promotion Guide for best-practice recommendations for promoting your work at: www.wileyauthors.com/eeo/guide. You can learn more about Wiley Editing Services which offers professional video, design, and writing services to create shareable video abstracts, infographics, conference posters, lay summaries, and research news stories for your research at: www.wileyauthors.com/eeo/promotion.

• If you would like to receive our 'Research Roundup', a monthly newsletter highlighting the cutting-edge research published

in The Physiological Society's family of journals (The Journal of Physiology, Experimental Physiology, Physiological Reports, The Journal of Nutritional Physiology and The Journal of Precision Medicine: Health and Disease), please click this link, fill in your name and email address and select 'Research Roundup':
https://www.physoc.org/journals-and-media/membernews

EDITOR COMMENTS

Reviewing Editor:

Given the revisions made by the authors, the reviewers and editors are now satisfied that their concerns have been addressed.

REFEREE COMMENTS

Referee #1:

I appreciate the authors' comprehensive responses to my comments and the significant revisions to the manuscript. These changes have enhanced the paper by moderating some claims, broadening the discussion of alternative neural pathways, clarifying the interpretation of latency results, and offering more detail on the statistical analyses and participant exclusions.

I appreciate that the authors have substantially softened their interpretation of the reticulospinal contribution throughout the Abstract, Key Points, and Discussion. Presenting the findings as indirect evidence consistent with brainstem, and likely reticulospinal, involvement is now much better aligned with the experimental evidence.

Overall, I think the manuscript now offers a well-balanced interpretation of a compelling and technically sound study. The experimental findings are innovative, the physiological questions are significant, and this work should encourage further research into how brainstem pathways influence human locomotor control.
